# Modelling and *in vitro* testing of the HIV-1 Nef fitness landscape

**DOI:** 10.1093/ve/vez029

**Published:** 2019-08-05

**Authors:** John P Barton, Erasha Rajkoomar, Jaclyn K Mann, Dariusz K Murakowski, Mako Toyoda, Macdonald Mahiti, Phillip Mwimanzi, Takamasa Ueno, Arup K Chakraborty, Thumbi Ndung’u

**Affiliations:** 1 Departments of Chemical Engineering, Physics, and Chemistry, Institute for Medical Engineering & Science, Massachusetts Institute of Technology, Cambridge, MA, USA; 2 Ragon Institute of Massachusetts General Hospital, Massachusetts Institute of Technology and Harvard University, Boston, MA, USA; 3 HIV Pathogenesis Programme, Doris Duke Medical Research Institute, Nelson R. Mandela School of Medicine, University of KwaZulu-Natal, Durban, South Africa; 4 Center for AIDS Research, Kumamoto University, Kumamoto, Japan; 5 International Research Center for Medical Sciences (IRCMS), Kumamoto University, Kumamoto, Japan; 6 Africa Health Research Institute, Durban, South Africa; 7 Max Planck Institute for Infection Biology, Chariteplatz, D-10117 Berlin, Germany

**Keywords:** fitness landscape, HIV Nef, HIV vaccine, HIV evolution, computational model

## Abstract

An effective vaccine is urgently required to curb the HIV-1 epidemic. We have previously described an approach to model the fitness landscape of several HIV-1 proteins, and have validated the results against experimental and clinical data. The fitness landscape may be used to identify mutation patterns harmful to virus viability, and consequently inform the design of immunogens that can target such regions for immunological control. Here we apply such an analysis and complementary experiments to HIV-1 Nef, a multifunctional protein which plays a key role in HIV-1 pathogenesis. We measured Nef-driven replication capacities as well as Nef-mediated CD4 and HLA-I down-modulation capacities of thirty-two different Nef mutants, and tested model predictions against these results. Furthermore, we evaluated the models using 448 patient-derived Nef sequences for which several Nef activities were previously measured. Model predictions correlated significantly with Nef-driven replication and CD4 down-modulation capacities, but not HLA-I down-modulation capacities, of the various Nef mutants. Similarly, in our analysis of patient-derived Nef sequences, CD4 down-modulation capacity correlated the most significantly with model predictions, suggesting that of the tested Nef functions, this is the most important *in vivo*. Overall, our results highlight how the fitness landscape inferred from patient-derived sequences captures, at least in part, the *in vivo* functional effects of mutations to Nef. However, the correlation between predictions of the fitness landscape and measured parameters of Nef function is not as accurate as the correlation observed in past studies for other proteins. This may be because of the additional complexity associated with inferring the cost of mutations on the diverse functions of Nef.

## 1. Introduction

Despite extensive research efforts, a vaccine or a cure for HIV-1 remains elusive. One of the major hurdles in vaccine design and development is eliciting a human immune response that is not evaded by the highly diverse and mutable HIV-1 (Kwong, Mascola, and Nabel 2012). Because HIV-1 fitness plays a significant role in disease progression (Deacon et al. 1995; Quiñones-Mateu et al. 2000; Song et al. 2012), continuous immune pressure on the wild-type virus favouring the outgrowth of escape variants with diminished fitness is a suggested vaccine strategy (Allen and Altfeld 2008; Chopera et al. 2011a). Although promising, this strategy poses a challenge because HIV-1 develops compensatory mutations that can restore its reduced fitness (Brockman et al. 2007; Crawford et al. 2007; Chopera et al. 2011a). Thus, mutations cannot be considered in isolation when assessing fitness consequences.

Our group has developed, and tested, computational models that aim to predict viral fitness based on the amino acid sequence and thereby quantify the relative fitness of strains containing different mutation patterns (Ferguson et al. 2013; Mann et al. 2014a; Barton et al. 2016b; Butler et al. 2016; Louie et al. 2018). These models may be used to identify deleterious mutational couplings with the end goal of potentially restricting viable escape of HIV-1 with vaccine immunogens that maximise sites that are harmful to HIV-1 when mutated in combination. We have previously applied this modelling approach to the HIV-1 Gag protein and validated the model by measuring the replication capacities of HIV-1 strains with various Gag mutation combinations *in vitro* (Ferguson et al. 2013; Mann et al. 2014a). It has also been successfully applied to study drug resistance mutations in HIV-1 protease (Butler et al. 2016), and to predict the process of escape in individual patients from T cell-mediated host immune pressure on diverse HIV proteins (Barton et al. 2016b).

Here we report a fitness landscape model of the HIV-1 Nef protein. Nef plays an important role in HIV-1 pathogenesis as well as host immune evasion (Kestler et al. 1991; Collins et al. 1998; Foster and Garcia 2008) making it an attractive vaccine target. Although Nef was included in the unsuccessful STEP trial, there is currently little evidence for or against its inclusion in vaccine strategies (Buchbinder et al. 2008). In this study, we evaluated and compared the accuracy of models of varying complexities in predicting the fitness consequences of mutations in HIV-1 Nef. In order to do so, we performed *in vitro* measurements of the Nef-driven replication capacities as well as Nef-mediated CD4 and HLA-I down-modulation capacities of thirty-two mutants, which were selected based on their predicted values of fitness derived from *in silico* models. Nef-mediated CD4 and HLA-I down-modulation capacities of the selected mutants were measured in addition to replicative fitness since these are key activities of the Nef protein that may influence pathogenesis (Iafrate et al. 2000; Swigut et al. 2004; Mann et al. 2014b). While CD4 down-modulation enhances viral replication through promoting the release of infectious viral particles (Ross, Oran, and Cullen 1999; Greenway et al. 2003), HLA-I down-modulation is not expected to directly influence HIV replication *in vitro* yet may still play a significant role in HIV-1 pathogenesis through enhancing immune evasion (Foster and Garcia 2008). Furthermore, we extended the testing of the model to patient-derived sequences of Nef, 298 subtype C Nef sequences (Mann et al. 2014b) and 150 subtype B Nef sequences (Mwimanzi et al. 2013a,b; Mahiti et al. 2015; Toyoda et al. 2015; Mahiti et al. 2016), for which several Nef functions were previously measured. For the subtype B Nef sequences, in addition to CD4 and HLA-I down-modulation, five other Nef activities were measured, namely down-modulation of CXCR4 and CCR5, upregulation of CD74, enhancement of virion infectivity, and enhancement of viral replication, allowing investigation of which Nef activities align most strongly with model predictions.

## 2. Materials and methods

### 2.1 Ising and Potts model inference

In order to infer the Ising and Potts models, we downloaded multiple sequence alignments (MSAs) of 11,354 HIV-1 Nef subtype B sequences and 6,469 subtype C sequences, obtained from 3,300 and 1,656 unique individuals, respectively, from the Los Alamos National Laboratory HIV Sequence Database (www.hiv.lanl.gov). We then processed the sequence data and employed the selective cluster expansion method (Cocco and Monasson 2011, 2012; Barton et al. 2016a) to infer Ising and Potts models that capture the pattern of correlated mutations observed in the sequence data, as previously described (Barton et al. 2016b); for completeness, methods for processing sequence data and information about the model inference are summarised in the Supplementary Material. Separate models are inferred for subtypes B and C data. In this model, the estimated prevalence *P*(***z***) of an HIV sequence ***z***, represented as a vector of amino acids, is given by

(1) $$P(z)=\frac{\exp\left(-E(z)\right)}{Q},\;E(z)=-\sum_{i=1}^{N}h_{i}(z_{i})-\sum_{i=1}^{N-1}\sum_{j=i+1}^{N}J_{\mathrm{ij}}(z_{i},z_{j})\;.$$

Here *N *=* *206 is the length of the protein, and the ***z***_*i*_ represent the amino acids present at each residue *i*. The parameters *h_i_*(***z***_*i*_) and *J_ij_*(***z***_*i*_, ***z***_*j*_), referred to as fields and couplings, are chosen so that the model reproduces the observed single- and double-mutant frequencies. Note that the values of *E*, referred to as energy, in Equation (1) are inversely related to prevalence, such that sequences with high *E* values have low prevalence, and vice versa. In past work, based on HIV biology, its evolutionary history and physics-based models, we have argued that, for HIV, the order of the prevalence of circulating strains is statistically similar to its fitness (i.e. ability to propagate infection) (Butler et al. 2016; Chakraborty 2017; Chakraborty and Barton 2017). This is in part due to HIV’s status as a chronic infection, its high mutation and recombination rates, and the great diversity of largely ineffective immune responses against it.

We considered models that account for the diversity of amino acids at each residue (Potts models) with a wide range of complexities, as measured by the entropy threshold *S_T_*, for selecting how many amino acids to include explicitly in the model at each residue. The possible values of the entropy threshold range from *S_T_* = 0 (the Ising model), where only the consensus amino acid at each residue is explicitly modelled and all other amino acids are treated as the same ‘mutant’ type, to *S_T_* = 1, where all observed amino acids are modelled explicitly. Here we inferred models with *S_T_* = 0, 0.5, 0.55, 0.6, 0.65, 0.7, 0.75, 0.8, 0.85, and 0.9. Overall agreement between the *E* values for all models was excellent (Supplementary Fig. S1). We use models trained on sequence data from the same HIV-1 subtype as in the experiment for the comparisons with experimental measurements reported below.

### 2.2 Analysis of mutant Nef sequences

#### 2.2.1 *Mutation selection*

To evaluate and compare the ability of the Ising and the Potts models to predict the fitness consequences of mutations in HIV-1 Nef, several mutations and mutation combinations were selected for testing based on their computed values of *E*. The models assign a value of *E* to each viral strain, which is predicted to be inversely related to the replicative fitness of the strain (Berg, Willmann, and Lässig 2004; Sella and Hirsh 2005; Ferguson et al. 2013; Mann et al. 2014a). Mutants were selected subjectively so that they covered the full range of *E* values. We included mutations in known functional motifs involved in CD4 and HLA-I down-modulation for comparison to what has been documented in literature. We also included HLA-associated mutations (which are likely to be cytotoxic T lymphocyte [CTL] escape mutations and are identified by statistical association with HLA alleles; Carlson et al. 2008) or known CTL escape mutations that covered a range of *E* values, since it would be desirable to identify CTL escape mutations with functional costs. Different amino acid mutations at the same codon with differential *E* values were also chosen to test the ability of Potts models to distinguish between different substitutions at the same codon. All mutations and their *E* values computed by the Ising and Potts models are summarised in Table 1.

**Table 1.** Energies, predicted by the Ising and Potts models, of the selected mutants.

Mutant^a^	Ising *E* (*S_T_* = 0)^b^	Potts *E* (*S_T_* = 0.9)^b^	Required for down-modulation of	HLA-association/known CTL escape^c^
17K19K	6.45	7.54	HLA and to a lesser extent CD4 (Schaefer et al. 2008)	
21E^d^	0.89	2.57		
21K^d^	0.89	1.55		
28E	0.22	0.51		C*08:02
33A	0.72	0.65		A*68:01; 33V is a known escape mutant (Goonetilleke et al. 2009)
43L	1.67	1.89		43V with C*03
33A43L	2.02	2.11		Pair of HLA-associated mutations
57G^d^	4.63	6.25	CD4 (Grzesiek et al. 1996; Mangasarian et al. 1999; Geyer, Fackler, and Peterlin 2001)	
57R^d^	4.63	5.53	CD4 (Grzesiek et al. 1996; Mangasarian et al. 1999; Geyer, Fackler, and Peterlin 2001)	
57R58P	7.44	8.32	CD4 (Grzesiek et al. 1996; Mangasarian et al. 1999; Geyer, Fackler, and Peterlin 2001)	
71K	1.63	1.81		C*07:02; 71T with B*07:02; 71T and 71R are known escape mutants (Ueno et al. 2007; Mwimanzi et al. 2011; Liu et al. 2013)
72L75L	11.75	12.77	HLA (Mangasarian et al. 1999; Foster et al. 2011)	
76V	3.51	3.65		B*81; C*18:01; 76V, 76T, 76I are known escape mutants (Leslie et al. 2006)
71K76V	5.14	5.47		Pair of HLA-associated mutations
80N^d^	3.58	4.30		B*07:02
76V80N	7.10	7.95		Pair of HLA-associated mutations
80D^d^	3.58	4.37		B*35:01; C*07:02
88G	3.99	4.32		Known escape mutant (Chopera et al. 2011b)
43L88G	5.63	6.17		Pair of HLA-associated mutations
102H^d^	0.31	0.60		B*44:03; C*08
28E102H^d^	0.61	1.17		Pair of HLA-associated mutations
102W^d^	0.31	1.85		
28E102W^d^	0.61	2.41		
123G	6.05	6.84	HLA and CD4 (Geyer, Fackler, and Peterlin 2001; Foster et al. 2011)	
133T	−0.11	0.47		B*35:01; 133I with B*38:01 and B*57
135F	1.15	1.20		A*23:01; A*24. A known escape mutant (Fujiwara et al. 2008)
133T135F	0.64	1.20		Pair of HLA-associated mutations
143Y	2.93	2.90		A*23:01
135F143Y	4.16	4.18		Pair of HLA-associated mutations
188H	1.34	2.40		A*31:01; 188R with B*58:01; 188S with A*30:01; 188N is a known escape mutant (Goonetilleke et al. 2009)
192R	1.45	1.74		192K with A*74. A known escape mutant (Kemal et al. 2008)
188H192R	2.40	3.82		Pair of HLA-associated mutations

a All mutants chosen represent the most common mutation at the corresponding residue with the exception of the additional mutations chosen to test the ability of the Potts model to distinguish between different amino acids at the same codon.

b The energies were computed for the mutations in the consensus B sequence background, where the sequence differences (A15T, T51N, C163S, Q170L, and K178R) from the multiple sequence alignment (MSA) consensus sequence were considered as additional mutations.

c Lists of HLA-associated polymorphisms in Nef were derived from Carlson et al. (2012, 2014). Mutations which are known CTL escape are referenced.

d Mutations chosen to test the ability of the Potts model to distinguish between different amino acids at the same codon.

#### 2.2.2 *Site-directed mutagenesis*

The mutations listed in Table 1 were introduced using the QuikChange II XL Site-Directed Mutagenesis kit (Stratagene, La Jolla, CA, USA) into the consensus B *nef* sequence (2004 consensus sequence available from the Los Alamos National Laboratory HIV Sequence Database [LANL]), which was first cloned into a TOPO vector (Invitrogen, Carlsbad, CA, USA). The procedure involved the generation of mutants through polymerase chain reaction (PCR) amplification of the desired template with custom designed mutagenic primers. The mutant plasmid was then transformed into XL10-Gold ultracompetent cells and sequencing was performed to confirm the presence of the correct mutation in *nef*-containing colonies. Following successful introduction of mutations, the mutant *nef* sequences were cloned into an HIV-1 NL4-3 plasmid. Briefly, an SF2 *nef* NL4-3 plasmid (containing NcoI and NotI restriction sites flanking SF2 *nef*), which was previously prepared (Fackler et al. 2006; Ueno et al. 2008), was digested with NcoI and NotI enzymes and the resulting *nef*-deleted NL4-3 plasmid was gel purified. PCR was performed to amplify the mutant *nef* sequences using forward and reverse primers containing the NcoI and NotI restriction sites, respectively. PCR products were digested with NcoI and NotI enzymes and then ligated to the digested *nef*-deleted NL4-3 plasmid using T4 ligase. The ligation mixture was transformed into XL10-Gold ultracompetent cells. Plasmids were then purified using a Qiagen maxiprep kit and sequenced to verify the correct mutant sequence.

#### 2.2.3 *Generation of mutant viruses in reporter cell lines and measurement of their replication capacities following virus infection of primary cells*

Mutant viruses were generated from the mutant *nef* NL4-3 plasmids as previously described (Wright et al. 2012). Briefly, this was carried out by electroporating 4 million green fluorescent protein (GFP)-reporter CEM-GXR T cells (described in Brockman et al. 2006; and obtained from Prof. Mark Brockman from Simon Fraser University) with 10 μg mutant plasmid. This resulted in production of mutant virus from the cells transfected with mutant plasmids. The percentage infected cells (GFP-positive cells) was monitored by flow cytometry and mutant viruses were harvested at 30 per cent infection. Replication capacities of the mutant viruses were measured in peripheral blood mononuclear cells (PBMCs) from two different HIV-1 negative donors as previously described (Ueno et al. 2008). Briefly, 1 million PBMCs were infected with mutant viruses at 7.5 ng p24 (pre-determined by p24 ELISA using the Retro-trek HIV-1 p24 Antigen ELISA 2.0 kit [ZeptoMetrix corporation, New York, NY, USA]), and were equally divided into four wells (200 µl each) of a ninety-six-well plate. On Day 3 of incubation, 100 µl supernatant was removed and PBMCs were stimulated with 100 µl of 10 µg/ml phytohaemagglutinin, and thereafter every 3 days 100 µl supernatant was removed and replaced with medium containing 20 U/ml interleukin-2. p24 concentration on Day 12 of incubation, at which point the viruses had detectable replication but had not yet surpassed peak replication, was used as the measure of replication capacity. The replication capacities of the mutant viruses were expressed as the mean of quadruplicate assessments in each donor and normalised to that of the LANL consensus B Nef-NL4-3 wild-type virus.

#### 2.2.4 *CD4 and HLA-I down-modulation assay using mutant Nef sequences cloned into pSELECT plasmid*

Nef-mediated CD4 and HLA-I down-modulation assays were performed as previously described in a GXR cell line which expresses high levels of CD4 and HLA-A*02 (Brockman et al. 2006) (obtained from Prof. Mark Brockman of Simon Fraser University). Briefly, the mutant *nef* sequences were cloned, as previously described (Mann et al. 2013), into a zeocin-resistant GFP-expressing plasmid (pSELECT) as this allowed detection by flow cytometry of cells transfected with Nef clones. GXR cells (600,000) were transfected by electroporation with the Nef clones (8 µg). Following a 20-h incubation, the cells were stained with fluorochrome-conjugated antibodies which bind to CD4 (APC-labelled anti-CD4 antibody, BD Biosciences, San Jose, CA, USA) and HLA-A*02 (PE-labelled HLA-A*02 antibody, BD Biosciences) molecules to allow for measurement of these molecules by flow cytometry. The down-modulation capacities of the mutant Nef clones were indicated by median fluorescence intensity of CD4 or HLA-A*02 expression in GFP-positive cells relative to that of the positive (SF2 pSELECT) and negative (ΔNef pSELECT) controls. This value was then normalised to that of the LANL consensus B Nef which was included in every assay. Assays were performed in duplicate and the results averaged.

#### 2.2.5 *Correlation analysis for Nef mutants*

The relationship between the predicted fitness costs of the mutant viruses (expressed as *E* values) and the measured replication capacities of the mutant viruses was evaluated using Spearman’s rank correlation. The relationship between the *E* values and the measured Nef-mediated CD4 and HLA-I down-modulation capacities was similarly evaluated. A significance level of *P* < 5 × 10^−2^ was used for all statistical analyses. *E* values were not corrected for the influence of phylogeny.

### 2.3 Analysis of patient-derived Nef sequences

#### 2.3.1 *Functional analysis of patient-derived Nef sequences*

The testing of the relationship between model energies and Nef function was extended to previously published patient-derived Nef sequences of subtype C (*n* = 298) (Mann et al. 2014b) and subtype B (*n* = 150) (Mwimanzi et al. 2013a,b; Mahiti et al. 2015, 2016; Toyoda et al. 2015), with each sequence obtained from a different patient. The subtype C Nef sequences were derived from southern Africa and comprised 107 sequences from recently infected individuals and 191 sequences from chronically infected individuals. CD4 and HLA-I down-modulation activities were previously measured, using Nef sequences cloned into pSELECT plasmid as described above, for those Nef sequences (Mann et al. 2014b). The subtype B Nef sequences were derived from Boston, NY, and Berlin, Germany, and comprised ninety-one sequences from chronically infected individuals (forty-five of whom were elite controllers) and fifty-nine sequences from acute infection. Down-modulation of HLA-I, CD4, CXCR4, and CCR5, upregulation of CD74, enhancement of virion infectivity, and enhancement of viral replication was previously measured for these subtype B Nef sequences, as previously described (Mwimanzi et al. 2013a,b; Mahiti et al. 2015, 2016; Toyoda et al. 2015). Briefly, HLA-I down-modulation, upregulation of CD74, virion infectivity, and viral replication capacity were measured using NL4-3 recombinant viruses encoding the patient-derived subtype B Nef sequences (Mwimanzi et al. 2013a,b; Mahiti et al. 2015, 2016). The Nef sequences were first cloned into a *nef*-deleted NL4-3 plasmid, as described earlier for the Nef mutant viruses, and then the recombinant viruses were generated by the transfection of HEK-293T cells followed by harvest of culture supernatants 48 h later (Mwimanzi et al. 2013b). HLA-I down-modulation and CD74 upregulation were determined in a 721.221 cell line stably expressing HLA-A*24:02, by infecting these cells with recombinant virus (300 ng p24) and staining cells 48 h later with fluorescently labelled antibodies for these molecules followed by flow cytometry (Mahiti et al. 2015). Nef-mediated enhancement of virion infectivity was measured in TZM-bl cells by chemiluminescence detection following infection of these cells with recombinant virus (3 ng p24) (Mwimanzi et al. 2013b). Nef-mediated enhancement of viral replication was measured following infection of PBMCs with recombinant virus (Mwimanzi et al. 2013b), as described earlier for the mutant Nef viruses. CD4, CXCR4, and CCR5 down-modulation were measured by transfecting Nef clones (patient-derived Nef sequences were cloned into the pIRES2-EGFP vector) into TZM-bl cells and subsequently detecting expression of these molecules by flow cytometry using fluorescently labelled antibodies (Toyoda et al. 2015). Notably, the CD4 measurements for the same subtype B Nef clones obtained in the TZM-bl cell line concurred well (*r* = 0.9 and *P* < 0.0001) with those obtained in a CEM T cell line (Mwimanzi et al. 2013a,b), as was used for CD4 down-regulation measurements for the mutant Nef and subtype C patient-derived Nef clones.

#### 2.3.2 *Correlation analysis for patient-derived Nef sequences*

Past work has shown a close relationship between the value of *E* in Equation (1) and fitness for a variety of HIV proteins (Ferguson et al. 2013; Mann et al. 2014a; Barton et al. 2016b; Louie et al. 2018). However, as mentioned above, contributions to fitness are more difficult to experimentally assess for a multifunctional protein such as Nef, especially since some of its functions may only be important *in vivo*. Furthermore, the existence of multiple functions that contribute to fitness makes comparisons between individual functional measurements and *E* more challenging. This is because *E*, which is inferred from diverse patient-derived sequences, is a proxy for *in vivo* fitness and concatenates the effects of mutations on the different Nef functions (including the unknown weights of each function *in vivo*). For example, consider a Nef protein that is unable to perform one of its critical functions (e.g. CD4 down-modulation), but where other, independent functions remain intact. In principle, because of this critical defect we would expect the *E* value of the corresponding sequence to be high. This makes sense in the context of the one function that is impaired, but not for the other Nef functions, which may appear normal. In short, high *E* values would lead us to expect low fitness and therefore functional impairment, but it need not be true that *all* functions are impaired in order for fitness to be affected. Thus, for a multifunctional protein, univariate correlations between *E* and individual functional measurements may not be strong even if *E* is a good predictor of overall fitness.

Thus, in order to develop a more complete picture of the potential contributions of different Nef functions to fitness, we used Nef functional measurements to predict the *E* value of the corresponding sequence with multiple linear regression. Assuming that *E* values are a reasonable proxy for fitness *in vivo*, this analysis would allow us to determine the extent to which impairment of each of the functions measured here contributes to fitness. In addition, we measured the correlation between each functional measurement and *E* as described above, but bearing in mind that these individual measurements do not reflect the complete picture.

## 3. Results

### 3.1 Analysis of mutant Nef sequences

#### 3.1.1 *Ising and Potts model predictions correlate with replication capacities of mutant viruses*

**Figure 1.**
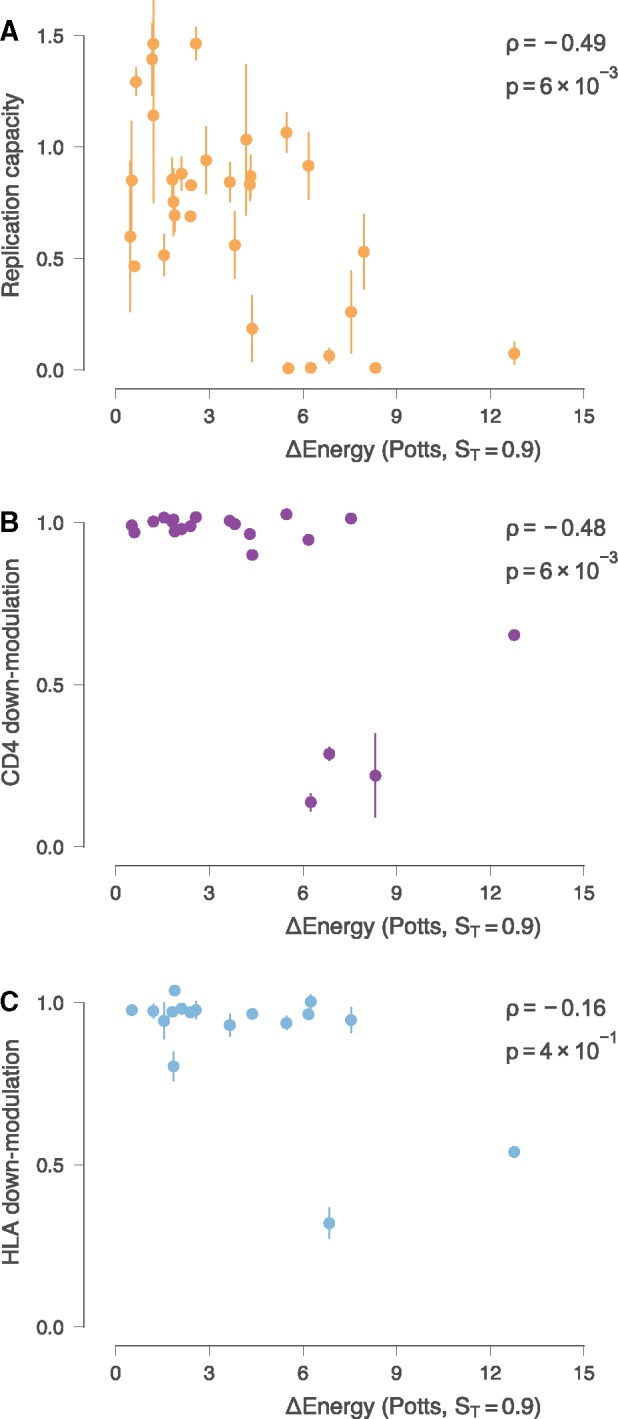
E values are negatively correlated with *in vitro* functional capacity of mutant viruses. Error bars in all panels denote the range of values obtained from experimental replicates. (A) Spearman rank correlation between replication capacities (RC) of mutant viruses and Potts model *E* values (*S_T_* = 0.9). RC of the mutant viruses is normalised relative to the wild-type virus. (B) Spearman rank correlation between CD4 down-modulation capability and *E* values. (C) Spearman rank correlation between HLA down-modulation capability and *E* values. The correlation between HLA down-modulation capability and *E* values is weak, but few mutant Nef sequences display significant impairment of HLA down-modulation.

The replication capacities of the Nef mutants were determined by infection of PBMCs followed by supernatant p24 ELISA measurement, and were normalised to the wild-type virus (Table 2). The experiments were performed in duplicate, using PBMCs from two different donors, and the results were averaged (concordance between replicates: Pearson *r* = 0.78, *P* = 3 × 10^−7^; Supplementary Fig. S2). *E* values for all models correlated significantly with the mutant replication capacities (Fig. 1A). The correlation did not depend on model complexity, that is, *S_T_* (Supplementary Fig. S3). Indeed, pairwise comparisons across models suggest that all differences in correlations fall short of the threshold of statistical significance (Supplementary Table S1). In the subsequent analysis we therefore present only correlations for the Potts model with *S_T_* = 0.9, which we have employed in past work (Mann et al. 2014a; Barton et al. 2016b). Results for models with other values of *S_T_* are similar in all cases. Full results are included in the Supplementary Material. Moreover, independent site forms of the model (i.e. those that include only the terms *h_i_*(***z***_*i*_) in Equation (1)) display similar levels of correlation (see Supplementary Fig. S4). This is somewhat surprising and is in contrast with previous work on other HIV proteins, which showed that models with couplings outperformed simple entropy (Ferguson et al. 2013; Mann et al. 2014a; Barton et al. 2016b) or models without couplings (Louie et al. 2018) for diverse HIV proteins. Furthermore, couplings were shown to make a more substantial effect on the dynamics of escape (Barton et al. 2016b), compared to static metrics of fitness measured *in vitro*. This is because of the high mutability and replication rates of HIV, which allows the virus to sample rare compensatory pathways dynamically. Such rare, coupled mutations may be uncommon in our data outside of T cell epitopes, which would contribute a small fraction to the energy overall. Past studies of bacterial proteins have also found an advantage to the inclusion of couplings for functional predictions (Figliuzzi et al. 2016). One reason for this may be that the Nef sequences in the patient-derived samples have mutations in few pairs of sites with strong couplings, or that the cumulative effect of the fields overwhelms the effects of couplings for sequences that are more phylogenetically distant (see Supplementary Fig. S5). Another may be that the multiple functions of Nef obscure the effect of couplings when we compare with *in vitro* assays of one function at a time.

**Table 2.** *In vitro* measurements of the replication capacities, CD4 down-modulation capacities and HLA-I down-modulation capacities of the thirty-two selected mutants.

Mutant	Ising *E* (*S_T_* = 0)	Potts *E* (*S_T_* = 0.9)	Replication capacity^a^	CD4 down-modulation^a^	HLA-I down-modulation^a^
17K19K	6.45	7.54	0.261	1.013	0.944
21E	0.89	2.57	1.464	1.017	0.977
21K	0.89	1.55	0.515	1.014	0.961
28E^b^	0.22	0.51	0.851	0.991	0.977
33A^b^	0.72	0.65	1.293	1.002	1.005
43L^b^	1.67	1.89	0.694	0.972	1.038
33A43L^b^	2.02	2.11	0.881	0.980	0.982
57G	4.63	6.25	0.009	0.138	1.003
57R	4.63	5.53	0.008	0.511	1.017
57R58P	7.44	8.32	0.001	0.220	1.026
71K^b^	1.63	1.81	0.854	1.002	0.972
72L75L	11.75	12.77	0.075	0.653	0.540
76V^b^	3.51	3.65	0.843	1.006	0.931
71K76V^b^	5.14	5.47	1.065	1.026	0.937
80N^b^	3.58	4.30	0.832	0.965	1.006
76V80N^b^	7.10	7.95	0.531	0.966	0.868
80D^b^	3.58	4.37	0.186	0.900	0.966
88G^b^	3.99	4.32	0.870	0.997	0.941
43L88G^b^	5.63	6.17	0.916	0.947	0.964
102H^b^	0.31	0.60	0.466	0.970	0.887
28E102H^b^	0.61	1.17	1.394	No result^c^	No result^c^
102W	0.31	1.85	0.754	1.008	0.804
28E102W	0.61	2.41	0.829	1.002	0.986
123G	6.05	6.84	0.064	0.287	0.320
133T^b^	−0.11	0.47	0.599	0.994	0.907
135F^b^	1.15	1.20	1.143	1.003	0.974
133T135F^b^	0.64	1.20	1.463	1.003	0.967
143Y^b^	2.93	2.90	0.941	0.978	0.990
135F143Y^b^	4.16	4.18	1.034	0.973	0.961
188H^b^	1.34	2.40	0.689	0.989	0.970
192R^b^	1.45	1.74	No result^c^	1.008	0.985
188H192R^b^	2.40	3.82	0.560	0.995	0.968

a Replication capacities, CD4 down-modulation capacities, and HLA-I down-modulation capacities are expressed relative to that of the wild-type LANL consensus B Nef.

b Escape or HLA-associated mutations (see Table 1 for further details).

c These results were unobtainable due to unsuccessful cloning of mutants 192R into the NL4-3 plasmid and 28E102H into the pSELECT plasmid.

Thus, our data can test our ability to predict the fitness landscape of HIV, but significantly more data would be required to test distinctions between the predictive capabilities of the different Potts models. Although good, the correlation between replication capacity and *E* was lower for Nef than in previous comparisons using Gag (Mann et al. 2014a) and Env (Louie et al. 2018) mutants, and the accuracy of estimates of viral evolution in individual patients using our estimated fitness landscape for other proteins (Barton et al. 2016b). This may be due to the complex behaviour of Nef *in vivo*, which is reflected in our fitness landscape inferred from *in vivo* data, only part of which is captured through *in vitro* assays of replication capacity. Consistent with this hypothesis, as we describe below, our data on CD4 down-modulation also correlates with predictions from the fitness landscape.

#### 3.1.2 *Model predictions are significantly correlated with CD4 down-modulation but not with HLA-I down-modulation capacities of mutant Nef sequences*

**Figure 2.**
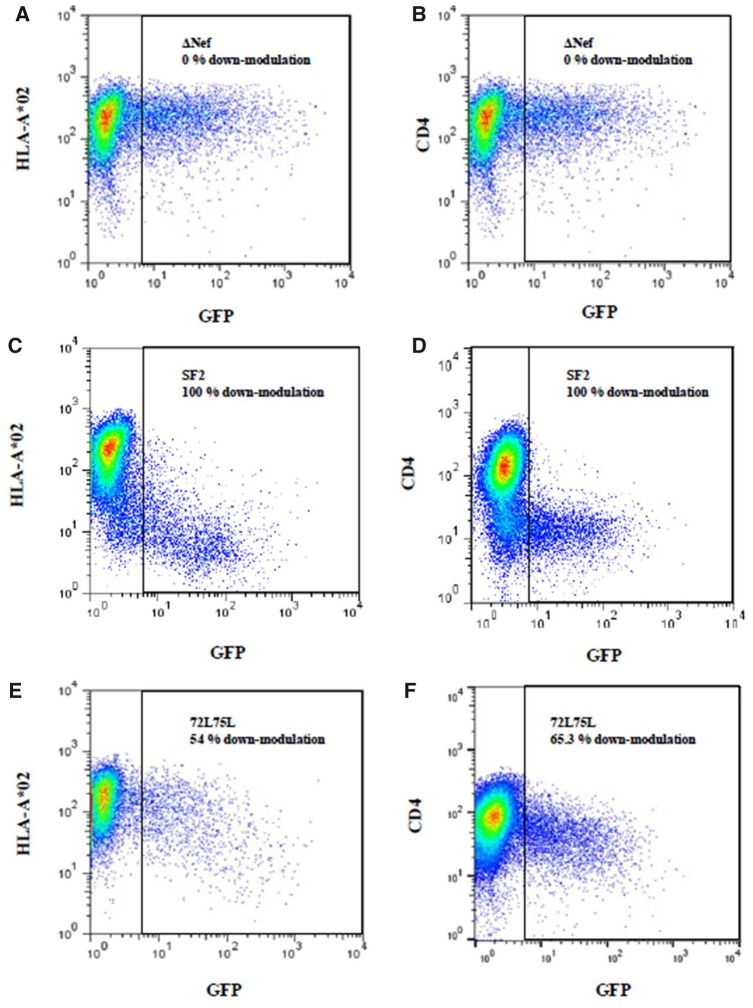
Representation of the flow cytometric measurements of Nef-mediated CD4 and HLA-I down-modulation. Graphs show the HLA-A*02 and CD4 cell surface expression on the *Y*-axis and green fluorescent protein (GFP) expression is indicated on the *X*-axis of graphs. GFP-positive cells represent cells successfully transfected with the Nef clones. HLA-A*02 and CD4 cell surface expression was measured in GFP-positive cells. (A, B) The HLA-A*02 (A) and CD4 (B) down-modulation ability of the negative control (ΔNef) is shown. The negative control represents 0 per cent down-modulation ability. (C, D) The HLA-A*02 (C) and CD4 (D) down-modulation ability of the positive control (SF2 Nef) is shown. The positive control represents 100 per cent down-modulation ability. (E, F) Graphs depict intermediate HLA down-modulation capacity (E) and CD4 down-modulation capacity by mutant 72L75L (F).

GXR cells expressing high levels of HLA-A*02 were transfected with the mutant Nef clones and cell surface expression of CD4 and HLA-A*02 in transfected cells was subsequently measured by flow cytometry. The CD4 and HLA-I down-modulation capacities of the mutant Nef clones are presented in Table 2. Representative flow cytometry plots are illustrated in Fig. 2.

**Figure 3.**
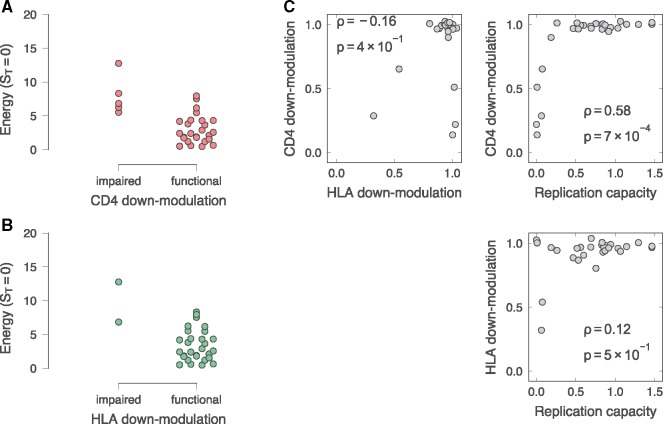
Relationships between *in vitro* Nef-mediated CD4 and HLA-I down-modulation capacities of mutant Nef clones and *E* values. (A) We find that viruses with impaired CD4 down-modulation capacities (<80% of reference) have significantly higher *E* values. (B) Viruses with impaired HLA-I down-modulation capacities also have higher *E* values, but this correlation falls below the threshold of significance. Nef-mediated CD4 and HLA-I down-modulation capacities of the mutant Nef clones were normalised to that of the wild-type LANL consensus B Nef. Here we show comparisons with Ising model (*S_T_* = 0) energies, which are consistent with results for more complex models (see Supplementary Fig. S3E and F). (C) Pairwise comparisons between replication capacity, CD4 down-modulation, and HLA-I down-modulation are shown, together with Spearman correlations. Note that all viruses with substantially impaired ability to down-modulate CD4 or HLA also have low replication capacities.

*E* values correlated significantly with the mutant CD4 down-modulation capacities (Spearman *ρ* = −0.48, *P* = 6 × 10^−3^; Fig. 1B), with little difference in correlation between model complexities (Supplementary Fig. S3). Substantial reductions in CD4 down-modulation are only observed at *E* values >5 (Fig. 3A). Further analysis showed that Nef mutant sequences with impaired ability to down-modulate CD4 (<0.8) have significantly higher energies than those that are competent at CD4 down-modulation (Mann–Whitney *U* = 7, *P* = 2 × 10^−3^), consistent with the above observation (Fig. 3A). There was no significant rank correlation between *E* values and the mutant HLA-I down-modulation capacities (Spearman *ρ* = −0.16, *P* = 4 × 10^−1^) (Fig. 1C), though this correlation is more difficult to evaluate due to the small number of Nef mutant sequences with significantly impaired ability to down-modulate HLA-I. Consistent with this, Nef mutant sequences with impaired ability to down-modulate HLA-I have higher energies than those that are competent, but near the threshold of significance (Mann–Whitney *U* = 3, *P* = 0.04) (Fig. 3B). As expected, CD4 down-modulation was correlated positively with replication capacity (*ρ* = 0.58 and *P* = 7 × 10^−4^; Fig. 3C) while no strong correlation was observed between HLA-I down-modulation and replication capacity (*ρ* = 0.12 and *P* = 5 × 10^−1^; Fig. 3C). However, due to the small sample of Nef mutant sequences with impaired ability to down-modulate CD4 and/or HLA-I, it is difficult to compare their importance based on this data alone. We explore this question in more detail in the analysis of a large sample of patient-derived sequences, described below.

#### 3.1.3 *CD4 and HLA-I down-modulation abilities of Nef mutant sequences are consistent with known functional motifs*

Several of the Nef mutations tested here (17K19K, 57G, 57R, 57R58P, 72L75L, and 123G) were in functional motifs previously described to be involved in either CD4 and/or HLA-I down-regulation (Table 1). We observed that Nef mutants 57G, 57R, 57R58P, 72L75L, and 123G had impaired ability to down-modulate CD4 (13.8–65.3% of wild-type levels). Accordingly, these mutants, with the exception of 72L75L, were previously noted in the literature to be important for CD4 down-modulation activity (Table 1). Also consistent with the literature, mutants 57G, 57R, and 57R58P retained the ability to down-modulate HLA (100–102% of wild-type levels) while CD4 down-modulation activity was lessened, and 123G was impaired for both CD4 and HLA down-modulation (28.7 and 32%, respectively). Mutations (17K19K, 72L75L, and 123G) in motifs previously shown to be required for HLA-I down-modulation (Table 1) also showed reduced HLA down-modulation relative to the wild-type virus (94.4, 54, and 32%, respectively), although for mutant 17K19K, HLA-I down-modulation was not substantially impaired. Therefore all mutations in the known functional motifs, with the exception of 17K19K, had substantially impaired function. These mutations, including the 17K19K, are predicted to have large values of *E* (*E* > 5, Table 1), consistent with experimental data.

### 3.2 HLA-associated/escape mutants tested did not significantly impair Nef function

A subset of HLA-associated mutations (likely cytotoxic T cell escape mutants) or known escape mutants, as well as pairs of these mutations, was selected that covered a range of *E* values (Table 1) as identification of escape mutants/pairs with substantial fitness costs would be useful for attenuation-based HIV vaccine design. Only one of the mutants in this category displayed a marked reduction in replication capacity, namely 80D (18.6% of wild-type levels), while the rest ranged from 46.6 to 146.3 per cent of wild-type levels (median of 85.1%) (Table 2). Overall, CD4 and HLA down-modulation capacities of mutants in this category were largely similar to wild-type (Table 2). The CD4 down-modulation capacities ranged from 90 to 102.6 per cent of wild-type levels (median of 99.4%) and HLA down-modulation capacities from 80.4 to 103.8 per cent of wild-type levels (median of 96.8%). This is in contrast to our previous study testing the models of the HIV-1 Gag fitness landscape where we identified several pairs of HLA-associated mutations with high *E* values that had substantial fitness costs i.e. viruses bearing these mutations did not replicate *in vitro* (Mann et al. 2014a). While there are HLA-associated mutations in Nef that reduce its function (Ueno et al. 2008; Kuang et al. 2014; Shahid et al. 2015), our results indicate that it is more difficult to identify escape mutations with a substantial fitness cost in this highly variable protein. Furthermore, considering that some of the HLA-associated mutations we studied here were previously reported to significantly impact Nef function (71K and 88G) (Mann et al. 2015; Naidoo et al. 2019) but had very moderate effects here in the LANL consensus subtype B background, the fitness cost of these mutations may be dependent on sequence background. However, in these results we emphasise that we have tested only a subset of HLA-associated mutations, not the comprehensive list of all escape or HLA-associated mutations in Nef.

#### 3.2.1 *Functional measurements of different amino acids at the same codon are in mixed agreement with* E *values predicted by the Potts model*

We explored whether the fitness outcome of different mutations at the same codon could be distinguished by Potts models. We tested the effect of different mutations at codons 21, 57, 80, 102 and codon combination 28/102 on Nef-driven replication capacity as well as Nef-mediated CD4 and HLA-I down-modulation ability (Table 2), and evaluated the consistency of results with the values of *E* (Tables 1 and 2) computed from Potts models where different mutant amino acids at these sites are modelled explicitly. These mutations were chosen as they represented examples of the most distinct Potts model *E* values for different pairs of amino acids at the same codon.

Mutants 57G and 57R both have similar, and very small, replication capacities, but the ability of 57G to down-modulate CD4 is substantially lower than for 57R. In agreement with this disparity, the Potts model assigns a higher *E* cost for the 57G mutation. Potts model predictions also successfully capture differences in fitness for 80D and 80N. Consistent with the higher *E* of 80D compared to 80N, the replication capacity of 80D is substantially lower than that of 80N. Next, we find that 102H has lower replication capacity than 102W, though the difference is not large compared to the variance in replication capacity measurements between experimental replicates. However, the HLA-I down-modulation ability of 102W is lower than that of 102H, and is in fact the third smallest among all Nef clones tested here. The Potts model predicts a higher fitness cost for 102W than for 102H, which is consistent with the HLA down-modulation data, but inconsistent with experimental replication capacities. The Potts model predicts higher fitness costs for 28E102W than for 28E102H, and in line with this the replication capacity of 28E102W was significantly lower than 28E102H. Finally, mutant 21E displays much higher replication capacity than 21K, with comparable CD4 and HLA-I down-modulation capabilities. However, the Potts model *E* cost of 21E is larger than 21K, and thus this difference is not correctly predicted by the model.

In summary, in three out of five instances the Potts model predictions were consistent with fitness/functional differences of different amino acid variants at the same codon. In one of the remaining two cases (102H versus 102W), the fitness/functional difference between the different amino acid variants is ambiguous, and for the other the model prediction is incorrect. In the case of 102H versus 102W, it is possible that the model captures a stronger influence of attenuated HLA-I down-modulation on *in vivo* fitness.

### 3.3 Analysis of patient-derived Nef sequences: CD4 down-regulation has the strongest connection with *E* values

Following our initial analysis of thirty-two closely-related Nef mutants, where each of the mutant sequences differed by only one or two amino acids from each other and a maximum of seven amino acids from the MSA, we next explored whether the model energies were also significantly related to the function of patient-derived Nef sequences which were considerably different from the constructed sequences and each other. Comparison between distant sequences is more challenging in part due to the influence of phylogeny, which affects prevalence (i.e. sequences closer to consensus are disproportionately likely to be observed). Prior work suggests that phylogeny particularly influences the inferred *h_i_* shown in Equation (1) (Shekhar et al. 2013). The effects of these biases on relative *E* values are anticipated to be small for closely-related sequences such as the collection of Nef mutants described above. However, for patient-derived Nef sequences that can differ by tens of mutations, phylogeny-induced shifts in the *E* values may make dominant contributions to the energy.

**Figure 4.**
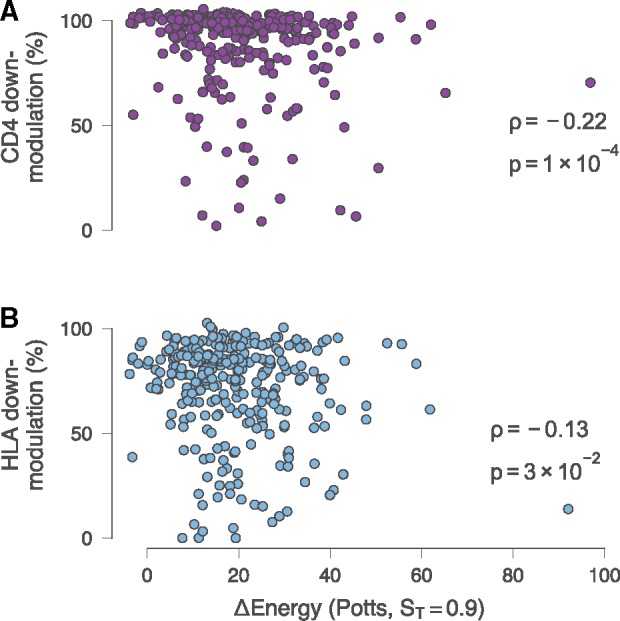
Relationships between *in vitro* Nef-mediated CD4 and HLA-I down-modulation capacities of natural subtype C Nef sequences and *E* values. (A) Energy values both Ising and Potts model energies are significantly negatively correlated with CD4 down-modulation. Note that energies shown here are derived from models trained on subtype C sequence data. (B) Correlation between energies and HLA-I down-modulation capacities are weaker than for CD4 down-modulation.

Using 298 subtype C Nef sequences (Mann et al. 2014b), as for the mutant Nef sequences, we observed a statistically significant correlation between the model energies and CD4 down-modulation activities of these patient-derived sequences (*ρ* *=* −0.22, *P* = 1.5 × 10^−4^) (Fig. 4A), although this was a weaker correlation than that observed with the mutant sequences. Interestingly, we found a weaker correlation between the model energies and HLA-I down-modulation ability that was not consistently statistically significant (*ρ* = −0.13, *P* = 2.7 × 10^−2^) (Fig. 4B). A limitation of the analyses presented for the mutant Nef sequences and subtype C Nef sequences is that only two to three Nef activities were measured, albeit ones suggested to significantly influence clinical outcome (Iafrate et al. 2000; Swigut et al. 2004; Mwimanzi et al. 2012; Mann et al. 2014b), while Nef displays a multitude of activities that may influence pathogenesis.

However, a more comprehensive functional analysis was previously performed on 150 subtype B patient-derived Nef sequences, for which 7 different Nef activities were analysed (Mwimanzi et al. 2013a,b; Mahiti et al. 2015, 2016; Toyoda et al. 2015). Here we focussed in particular on the subset of ninety-one sequences for which all seven Nef functional measurements were performed. First we measured the relationship between *E* and each Nef function individually (Fig. 5). We found that *E* was most significantly negatively correlated with CD4 down-modulation (*ρ* = −0.30, *P* = 4.1 × 10^−3^), followed by CCR5 down-modulation (*ρ* = −0.18, *P* = 8.8 × 10^−2^) and CXCR4 down-modulation (*ρ* = −0.13, *P* = 2.1 × 10^−1^).

However, as detailed in Section 2, such individual comparisons can be misleading because, assuming that *E* is a good proxy for fitness, a defect in one important Nef function may lead us to infer a high *E* for the corresponding sequence even though other Nef functions remain intact. We thus employed multiple linear regression to gain insight into the overall relationship between *E* and Nef function, using Nef functional measurements to estimate the *E*. This also allows us to roughly quantify how much each Nef function contributes to *in vivo* fitness: although *E* is only an estimate it represents the totality of Nef function, and our goal was to determine which of the components measured would align more closely (and therefore likely contribute most) to the sum of Nef function. Here we normalised values across functional measurements by converting them to *z*-scores. Using multiple linear regression, we found that overall these seven Nef functions could explain approximately 25 per cent of the variance in energies (*R*^2^ = 0.26, adjusted *R*^2^ = 0.19, *P* = 6.2 × 10^−4^; see Table 3). CD4 down-modulation had the largest and most significant association with *E*, suggesting that this function of Nef is particularly important *in vivo*. We find similar results using quantile regression, a form of linear regression that aims to minimise total absolute residuals, thereby being more robust to outliers (see Supplementary Table S2).

**Table 3.** Multiple linear regression of normalised functional measurements against Ising and Potts model E for natural subtype B sequences.

Predictor	Coefficient (Ising)	*P*-value (Ising)	Coefficient (Potts, *S_T_* = 0.9)	*P*-value (Potts, *S_T_* = 0.9)
CD4 down-modulation	−0.492	0.002	−0.549	0.001
HLA down-modulation	−0.015	0.895	0.061	0.586
CXCR4 down-modulation	−0.111	0.452	−0.049	0.739
CCR5 down-modulation	0.093	0.647	0.098	0.627
CD74 upregulation	0.266	0.022	0.269	0.020
Infectivity	−0.151	0.206	−0.181	0.130
Replication capacity	0.008	0.945	0.008	0.944

## 4. Discussion

In this study we inferred computational models of the HIV-1 Nef fitness landscape and firstly evaluated the models by testing predictions against *in vitro* measurements of Nef-driven replication capacities as well as the Nef-mediated CD4 and HLA-I down-modulation capacities of thirty-two different Nef mutants. We observed that predictions from models of varying complexities correlated significantly with Nef-driven replication capacities and CD4 down-modulation capacities, but not HLA-I down-modulation capacities, of the various Nef mutants: mutations with higher values of *E* tended to show lower Nef-driven replication as well as costs to CD4 down-modulation capacity. However, these correlations were weaker than those previously observed between comparisons of fitness landscape-based predictions for other HIV proteins and *in vitro* and clinical data (Ferguson et al. 2013; Mann et al. 2014a; Barton et al. 2016b; Butler et al. 2016; Louie et al. 2018). For example, correlations of *r* = −0.81 (Ferguson et al. 2013) and *r* = −0.83 (Mann et al. 2014a) were previously observed between measured HIV Gag-driven replication capacity and *E* and similar results (*r* = −0.74) were also found for HIV Env in recent work (Louie et al. 2018). This difference may be due in part to the more complex way in which Nef contributes to HIV-1 fitness *in vivo* (Foster and Garcia 2008), which is challenging to comprehensively measure through *in vitro* tests. It is important to note that, because our fitness model is constructed from sequences derived from patients, it reflects a concatenation of multiple Nef functions (Foster and Garcia 2008). This presents a challenge in using our approach to predict which specific Nef functions of a particular sequence might be impaired because selection for multiple functions contributes to the distribution of sequences that are observed in clinical data. Furthermore these pressures on the different functions of Nef may fluctuate during disease progression (Carl et al. 2001; Lewis et al. 2008). Our model also neglects intergenic epistasis, which could be one additional source of variance especially for the widely-diverged natural Nef sequences which were cloned into the same viral background for experimental measurements. Prior work has estimated that intergenic epistasis is small for HIV (Hinkley et al. 2011), but we cannot rule out its influence. It is also true that our inferred fitness landscape can only be expected to be correct in a statistical way, not necessarily precise for every case. Consistent with this, the experimental data presented here are significantly correlated with model predictions, indicating that the models were able to predict fitness costs in the Nef protein better than chance. Importantly, we observed that the functional experimental outcomes for mutations within known functional motifs involved in Nef-mediated CD4 and/or HLA-I down-modulation were consistent with what was previously documented in the literature, supporting the validity of our functional measurements.

Furthermore, we extended our evaluation of the models to patient-derived sequences of Nef, 298 subtype C Nef sequences (for which CD4 and HLA-I down-modulation capacities were measured) (Mann et al. 2014b) and 150 subtype B Nef sequences (for which 7 different Nef activities were measured) (Mwimanzi et al. 2013a,b; Mahiti et al. 2015, 2016; Toyoda et al. 2015), which were far apart in sequence space from each other and the MSA from which the landscape was inferred. Nevertheless, we still observed a significant inverse correlation between CD4 down-modulation capacity of these patient-derived Nef sequences and model energies. The subtype B patient-derived Nef sequences represented a more comprehensive analysis of Nef function. In combination, the seven Nef activities significantly correlated with the model, although only CD4 down-modulation, followed by CXCR4/CCR5 down-modulation (at borderline significance), correlated individually, and CD4 down-modulation capacity contributed the most to the correlation in the multiple linear regression. Thus, in all analyses of mutant and patient-derived Nef sequences, CD4 down-modulation emerged as the Nef activity most strongly aligned with model predictions, suggesting that this Nef activity is the strongest determinant (out of the Nef activities measured here) of the *in vivo* effect of Nef, although we cannot rule out that other Nef activities not measured here, known or unknown, may be equally or more important. In support of this, in previous work when a Nef motif essential for CD4 down-modulation was disrupted (yet HLA down-modulation function was intact) simian immunodeficiency virus replication was greatly reduced (Iafrate et al. 2000), and studies in the humanised mouse model indicated that CD4 down-modulation plus one or more unknown Nef activities contribute the most strongly to Nef’s pathogenic effect (Watkins et al. 2013).

It should be noted that there were some differences between the results of the mutant and patient-derived Nef analyses. For example, in the mutant analysis, HLA down-modulation capacity did not correlate significantly with the model energies; however, in the analysis of the subtype C patient-derived Nef sequences, it did, although not consistently for all model complexities. This discrepancy may be partly due to the fact that only two of the mutants displayed substantially reduced HLA-I down-modulation capacity, while there was a greater spread of values for this parameter in the patient-derived Nef sequences, which limited our ability to accurately assess correlations between HLA-I down-modulation and other measurements in the mutant group. The overall correlation values with individual functional measurements are also modest, and thus larger sample sizes are needed to observe them reliably.

The Potts model is expected to be more reliable than the Ising model in predicting the fitness costs of mutations in highly mutable proteins such as Nef since the Potts model has residue-specific resolution. The Ising model does not consider the identity of the mutant amino acid but rather uses a binary approximation (all mutant amino acids are denoted as one and the wild-type amino acid is denoted as zero) which we expected may not be sufficient for highly variable proteins. However, while the *E* values of all models correlated significantly with replication capacity and CD4 down-modulation of the mutant Nef sequences, differences between the models could not be determined in a statistically significant way (Supplementary Table S1). We also observed that the values of *E* assigned by the Potts model to different mutations at the same codon correctly reflected the functional/fitness consequences of these mutations in three out of five cases studied, was ambiguous in one case, and incorrect in one case.

In aggregate, we observe that the performance of the Ising and Potts models is similar. The surprising success of the Ising model may be because, in most cases, the most common mutation at a particular codon was tested. The binary approximation of the Ising model is expected to be more representative of the most common mutations rather than rare ones (Mann et al. 2014a). The difference between the expected and experimental outcomes might also be due in part to the inclusion of less well-sampled and thus noisier mutation information, in particular for the most complex models (*S_T_* > 0.8, where performance more clearly degrades). Essentially, while considering multiple amino acids provides more information about the sequence distribution, the negative effect of additional noise may potentially outweigh the gain in information. While our data may potentially caution against overly complex models, more extensive tests will be required in order to thoroughly test the association between model complexity and predictions of viral function.

Our study has additional limitations. Firstly, only the down-modulation of HLA-A*02 was measured for the mutant Nef sequences and the subtype C patient-derived sequences (while HLA-A*24:02 was measured for the subtype B patient-derived sequences). However, in a previous study Nef-mediated HLA-A*02 down-modulation was highly correlated with HLA-B*07 down-modulation (Spearman *ρ* = 0.89, *P* < 1 × 10^−4^) (Mann et al. 2013). Additionally, HLA down-modulation occurs through a sequence shared by the cytoplasmic tail of HLA-A and HLA-B molecules (Le Gall et al. 1998). Therefore, the ability of Nef to down-modulate HLA-A and HLA-B molecules may be generally represented by its ability to down-modulate HLA-A*02. However, it is possible that the ability of Nef to down-modulate HLA may differ depending on the HLA allele (Mahiti et al. 2016) and therefore it may be worthwhile to measure down-modulation of different HLA alleles. The second limitation was that even though Nef is involved in many cellular functions (Foster et al. 2011), we focussed only on replication capacity, CD4 down-modulation, and HLA-I down-modulation for the mutant Nef sequences. Using *in vitro* assays that only measure limited known functions of the multifunctional Nef protein, for which there may be yet unknown important functions, is a significant limitation for our study. However, while the functions we measured through our *in vitro* assays do not represent a comprehensive list, it was shown that they are important *in vivo* and do influence clinical outcome (Iafrate et al. 2000; Swigut et al. 2004; Mwimanzi et al. 2012; Mann et al. 2014b). Furthermore, this limitation was somewhat mitigated by our analysis of 150 subtype B patient-derived Nef sequences for which 7 different Nef activities were measured. Lastly, we utilised PBMCs, since the presence of Nef is not essential for viral replication in many immortalised T cell lines (Lundquist et al. 2002), but PBMCs are highly variable between donors (Brockman et al. 2006). Nevertheless, we measured Nef-driven replication capacities using two different donors in this study and the measurements of the two donors were in good agreement. Variance between replication capacity measurements in different donors does limit fine distinctions between replication capacities, however.

In conclusion, the experimental data was in significant agreement with predictions of the fitness consequences of mutations in the Nef protein, and with continuing validation efforts (Ferguson et al. 2013; Mann et al. 2014a; Barton et al. 2016b; Butler et al. 2016; Chakraborty 2017; Chakraborty and Barton 2017), these models can be used to direct immunogen design in a manner similar to that outlined for the Gag protein (Ferguson et al. 2013).

## Supplementary Material

vez029_Supplementary_Data

## References

[vez029-B1] Allen T. M., Altfeld M. (2008) ‘Crippling HIV One Mutation at a Time’, The Journal of Experimental Medicine, 205: 1003–7. 18458116 10.1084/jem.20080569 PMC2373833

[vez029-B2] Barton J. P. et al (2016a) ‘ACE: Adaptive Cluster Expansion for Maximum Entropy Graphical Model Inference’, Bioinformatics, 32: 3089–97. 27329863 10.1093/bioinformatics/btw328

[vez029-B3] Barton J. P. et al (2016b) ‘Relative Rate and Location of Intra-Host HIV Evolution to Evade Cellular Immunity Are Predictable’, Nature Communications, 7: 11660. 10.1038/ncomms11660 PMC4879252 27212475

[vez029-B4] Berg J., Willmann S., Lässig M. (2004) ‘Adaptive Evolution of Transcription Factor Binding Sites’, BMC Evolutionary Biology, 4: 42. 15511291 10.1186/1471-2148-4-42 PMC535555

[vez029-B5] Brockman M. A. et al (2006) ‘Use of a Novel GFP Reporter Cell Line to Examine Replication Capacity of CXCR4- and CCR5-Tropic HIV-1 by Flow Cytometry’, Journal of Virological Methods, 131: 134–42. 16182382 10.1016/j.jviromet.2005.08.003

[vez029-B6] Brockman M. A. et al (2007) ‘Escape and Compensation from Early HLA-B57-Mediated Cytotoxic T-Lymphocyte Pressure on Human Immunodeficiency Virus Type 1 Gag Alter Capsid Interactions with Cyclophilin A’, Journal of Virology, 81: 12608–18. 17728232 10.1128/JVI.01369-07 PMC2169025

[vez029-B7] Buchbinder S. P. et al (2008) ‘Efficacy Assessment of a Cell-Mediated Immunity HIV-1 Vaccine (the Step Study): A Double-Blind, Randomised, Placebo-Controlled, Test-of-Concept Trial’, The Lancet, 372: 1881–93. 10.1016/S0140-6736(08)61591-3 PMC2721012 19012954

[vez029-B8] Butler T. C. et al (2016) ‘Identification of Drug Resistance Mutations in HIV from Constraints on Natural Evolution’, Physical Review. E, 93: 022412. 26986367 10.1103/PhysRevE.93.022412

[vez029-B9] Carl S. et al (2001) ‘Modulation of Different Human Immunodeficiency Virus Type 1 Nef Functions during Progression to AIDS’, Journal of Virology, 75: 3657–65. 11264355 10.1128/JVI.75.8.3657-3665.2001 PMC114857

[vez029-B10] Carlson J. M. et al (2008) ‘Phylogenetic Dependency Networks: Inferring Patterns of CTL Escape and Codon Covariation in HIV-1 Gag’, PLoS Computational Biology, 4: e1000225. 19023406 10.1371/journal.pcbi.1000225 PMC2579584

[vez029-B11] Carlson J. M. et al (2012) ‘Widespread Impact of HLA Restriction on Immune Control and Escape Pathways of HIV-1’, Journal of Virology, 86: 5230–43. 22379086 10.1128/JVI.06728-11 PMC3347390

[vez029-B12] Carlson J. M. et al (2014) ‘HIV Transmission. Selection Bias at the Heterosexual HIV-1 Transmission Bottleneck’, Science (New York, N.Y.), 345: 1254031. 10.1126/science.1254031 PMC4289910 25013080

[vez029-B13] Chakraborty A. K. (2017) ‘A Perspective on the Role of Computational Models in Immunology’, Annual Review of Immunology, 35: 403–39. 10.1146/annurev-immunol-041015-055325 28226229

[vez029-B14] Chakraborty A. K., Barton J. P. (2017) ‘Rational Design of Vaccine Targets and Strategies for HIV: A Crossroad of Statistical Physics, Biology, and Medicine’, Reports on Progress in Physics, 80: 032601. 28059778 10.1088/1361-6633/aa574a

[vez029-B15] Chopera D. R. et al (2011a) ‘Immune-Mediated Attenuation of HIV-1’, Future Virology, 6: 917–28. 22393332 10.2217/fvl.11.68 PMC3292540

[vez029-B16] Chopera D. R. et al (2011b) ‘Virological and Immunological Factors Associated with HIV-1 Differential Disease Progression in HLA-B 58:01-Positive Individuals’, Journal of Virology, 85: 7070–80. 21613398 10.1128/JVI.02543-10 PMC3126593

[vez029-B17] Cocco S., Monasson R. (2011) ‘Adaptive Cluster Expansion for Inferring Boltzmann Machines with Noisy Data’, Physical Review Letters, 106: 90601. 10.1103/PhysRevLett.106.090601 21405611

[vez029-B18] Cocco S., Monasson R. (2012) ‘Adaptive Cluster Expansion for the Inverse Ising Problem: Convergence, Algorithm, and Tests’, Journal of Statistical Physics, 147: 252–314.

[vez029-B19] Collins K. L. et al (1998) ‘HIV-1 Nef Protein Protects Infected Primary Cells against Killing by Cytotoxic T Lymphocytes’, Nature, 391: 397–401. 9450757 10.1038/34929

[vez029-B20] Crawford H. et al (2007) ‘Compensatory Mutation Partially Restores Fitness and Delays Reversion of Escape Mutation within the Immunodominant HLA-B*5703-Restricted Gag Epitope in Chronic Human Immunodeficiency Virus Type 1 Infection’, Journal of Virology, 81: 8346–51. 17507468 10.1128/JVI.00465-07 PMC1951305

[vez029-B21] Deacon N. J. et al (1995) ‘Genomic Structure of an Attenuated Quasi Species of HIV-1 from a Blood Transfusion Donor and Recipients’, Science (New York, N.Y.), 270: 988–91. 10.1126/science.270.5238.988 7481804

[vez029-B22] Fackler O. T. et al (2006) ‘Functional Characterization of HIV-1 Nef Mutants in the Context of Viral Infection’, Virology, 351: 322–39. 16684552 10.1016/j.virol.2006.03.044

[vez029-B23] Ferguson A. L. et al (2013) ‘Translating HIV Sequences into Quantitative Fitness Landscapes Predicts Viral Vulnerabilities for Rational Immunogen Design’, Immunity, 38: 606–17. 23521886 10.1016/j.immuni.2012.11.022 PMC3728823

[vez029-B24] Figliuzzi M. et al (2016) ‘Coevolutionary Landscape Inference and the Context-Dependence of Mutations in Beta-Lactamase TEM-1’, Molecular Biology and Evolution, 33: 268–80. 26446903 10.1093/molbev/msv211 PMC4693977

[vez029-B25] Foster J. L., Garcia J. V. (2008) ‘HIV-1 Nef: At the Crossroads’, Retrovirology, 5: 84. 18808677 10.1186/1742-4690-5-84 PMC2563024

[vez029-B26] Foster J. L. et al (2011) ‘Mechanisms of HIV-1 Nef Function and Intracellular Signaling’, Journal of Neuroimmune Pharmacology : The Official Journal of the Society on Neuroimmune Pharmacology, 6: 230–46. 21336563 10.1007/s11481-011-9262-y PMC3777542

[vez029-B27] Fujiwara M. et al (2008) ‘Different Abilities of Escape Mutant-Specific Cytotoxic T Cells to Suppress Replication of Escape Mutant and Wild-Type Human Immunodeficiency Virus Type 1 in New Hosts’, Journal of Virology, 82: 138–47. 10.1128/JVI.01452-07 PMC2224353 17959671

[vez029-B28] Geyer M., Fackler O. T., Peterlin B. M. (2001) ‘Structure–Function Relationships in HIV-1 Nef’, EMBO Reports, 2: 580–5. 11463741 10.1093/embo-reports/kve141 PMC1083955

[vez029-B29] Goonetilleke N. et al (2009) ‘The First T Cell Response to Transmitted/Founder Virus Contributes to the Control of Acute Viremia in HIV-1 Infection’, The Journal of Experimental Medicine, 206: 1253–72. 19487423 10.1084/jem.20090365 PMC2715063

[vez029-B30] Greenway A. L. et al (2003) ‘HIV-1 Nef Control of Cell Signalling Molecules: Multiple Strategies to Promote Virus Replication’, Journal of Biosciences, 28: 323–35. 12734410 10.1007/BF02970151

[vez029-B31] Grzesiek S. et al (1996) ‘The CD4 Determinant for Downregulation by HIV-1 Nef Directly Binds to Nef. Mapping of the Nef Binding Surface by NMR’, Biochemistry, 35: 10256–61. 8756680 10.1021/bi9611164

[vez029-B32] Hinkley T. et al (2011) ‘A Systems Analysis of Mutational Effects in HIV-1 Protease and Reverse Transcriptase’, Nature Genetics, 43: 487–9. 21441930 10.1038/ng.795

[vez029-B33] Iafrate A. J. et al (2000) ‘Disrupting Surfaces of Nef Required for Downregulation of CD4 and for Enhancement of Virion Infectivity Attenuates Simian Immunodeficiency Virus Replication In Vivo’, Journal of Virology, 74: 9836–44. 11024110 10.1128/jvi.74.21.9836-9844.2000 PMC102020

[vez029-B34] Kemal K. S. et al (2008) ‘Transition from Long-Term Nonprogression to HIV-1 Disease Associated with Escape from Cellular Immune Control’, Journal of Acquired Immune Deficiency Syndromes (1999), 48: 119–26. 18520675 10.1097/QAI.0b013e31816b6abd

[vez029-B35] Kestler H. W. et al (1991) ‘Importance of the Nef Gene for Maintenance of High Virus Loads and for Development of AIDS’, Cell, 65: 651–62. 2032289 10.1016/0092-8674(91)90097-i

[vez029-B36] Kuang X. T. et al (2014) ‘Impaired Nef Function Is Associated with Early Control of HIV-1 Viremia’, Journal of Virology, 88: 10200–13. 24965469 10.1128/JVI.01334-14 PMC4136354

[vez029-B37] Kwong P. D., Mascola J. R., Nabel G. J. (2012) ‘The Changing Face of HIV Vaccine Research’, Journal of the International Aids Society, 15: 17407. 22789610 10.7448/IAS.15.2.17407 PMC3499796

[vez029-B38] Le Gall S. et al (1998) ‘Nef Interacts with the μ Subunit of Clathrin Adaptor Complexes and Reveals a Cryptic Sorting Signal in MHC I Molecules’, Immunity, 8: 483–95. 9586638 10.1016/s1074-7613(00)80553-1

[vez029-B39] Leslie A. et al (2006) ‘Differential Selection Pressure Exerted on HIV by CTL Targeting Identical Epitopes but Restricted by Distinct HLA Alleles from the Same HLA Supertype’, Journal of Immunology (Baltimore, Md.: 1950), 177: 4699–708. 10.4049/jimmunol.177.7.4699 16982909

[vez029-B40] Lewis M. J. et al (2008) ‘Functional Adaptation of Nef to the Immune Milieu of HIV-1 Infection in Vivo’, Journal of Immunology (Baltimore, Md.: 1950), 180: 4075–81. 10.4049/jimmunol.180.6.4075 18322217

[vez029-B41] Liu M. K. et al (2013) ‘Vertical T Cell Immunodominance and Epitope Entropy Determine HIV-1 Escape’, The Journal of Clinical Investigation, 123: 380–93. 23221345 10.1172/JCI65330 PMC3533301

[vez029-B42] Louie R. H. Y. et al (2018) ‘Fitness Landscape of the Human Immunodeficiency Virus Envelope Protein That Is Targeted by Antibodies’, Proceedings of the National Academy of Sciences of the United States of America, 115: E564–73. 29311326 10.1073/pnas.1717765115 PMC5789945

[vez029-B43] Lundquist C. A. et al (2002) ‘Nef-Mediated Downregulation of CD4 Enhances Human Immunodeficiency Virus Type 1 Replication in Primary T Lymphocytes’, Journal of Virology, 76: 4625–33. 11932428 10.1128/JVI.76.9.4625-4633.2002 PMC155097

[vez029-B44] Mahiti M. et al (2015) ‘Dynamic Range of Nef-Mediated Evasion of HLA Class II-Restricted Immune Responses in Early HIV-1 Infection’, Biochemical and Biophysical Research Communications, 463: 248–54. 25998395 10.1016/j.bbrc.2015.05.038

[vez029-B45] Mahiti M. et al (2016) ‘Relative Resistance of HLA-B to Downregulation by Naturally Occurring HIV-1 Nef Sequences’, mBio, 7: e01516-15. 10.1128/mBio.01516-15 PMC4724998 26787826

[vez029-B46] Mangasarian A. et al (1999) ‘Nef-Induced CD4 and Major Histocompatibility Complex Class I (MHC-I) Down-Regulation Are Governed by Distinct Determinants: N-Terminal Alpha Helix and Proline Repeat of Nef Selectively Regulate MHC-I Trafficking’, Journal of Virology, 73: 1964–73. 9971776 10.1128/jvi.73.3.1964-1973.1999 PMC104438

[vez029-B47] Mann J. K. et al (2013) ‘Ability of HIV-1 Nef to Downregulate CD4 and HLA Class I Differs among Viral Subtypes’, Retrovirology, 10: 100. 24041011 10.1186/1742-4690-10-100 PMC3849644

[vez029-B48] Mann J. K. et al (2014a) ‘The Fitness Landscape of HIV-1 Gag: Advanced Modeling Approaches and Validation of Model Predictions by In Vitro Testing’, PLoS Computational Biology, 10: e1003776. 25102049 10.1371/journal.pcbi.1003776 PMC4125067

[vez029-B49] Mann J. K. et al (2014b) ‘Nef-Mediated Down-Regulation of CD4 and HLA Class I in HIV-1 Subtype C Infection: Association with Disease Progression and Influence of Immune Pressure’, Virology, 468: 214–25. 25193656 10.1016/j.virol.2014.08.009 PMC4252354

[vez029-B50] Mann J. K. et al (2015) ‘Genetic Determinants of Nef-Mediated CD4 and HLA Class I Down-Regulation Differences between HIV-1 Subtypes B and C’, Virology Journal, 12: 200. 26607225 10.1186/s12985-015-0429-7 PMC4660847

[vez029-B51] Mwimanzi P. et al (2011) ‘Effects of Naturally-Arising HIV Nef Mutations on Cytotoxic T Lymphocyte Recognition and Nef's Functionality in Primary Macrophages’, Retrovirology, 8: 50. 21696586 10.1186/1742-4690-8-50 PMC3131245

[vez029-B52] Mwimanzi P. et al (2012) ‘Human Leukocyte Antigen (HLA) Class I Down-Regulation by Human Immunodeficiency Virus Type 1 Negative Factor (HIV-1 Nef): What Might We Learn from Natural Sequence Variants?’, Viruses, 4: 1711–30. 23170180 10.3390/v4091711 PMC3499827

[vez029-B53] Mwimanzi P. et al (2013a) ‘Dynamic Range of Nef Functions in Chronic HIV-1 Infection’, Virology, 439: 74–80. 23490051 10.1016/j.virol.2013.02.005

[vez029-B54] Mwimanzi P. et al (2013b) ‘Attenuation of Multiple Nef Functions in HIV-1 Elite Controllers’, Retrovirology, 10: 1. 23289738 10.1186/1742-4690-10-1 PMC3564836

[vez029-B55] Naidoo L. et al (2019) ‘Nef-Mediated Inhibition of NFAT following TCR Stimulation Differs between HIV-1 Subtypes’, Virology, 531: 192–202. 30927712 10.1016/j.virol.2019.02.011 PMC6526282

[vez029-B56] Quiñones-Mateu M. E. et al (2000) ‘A Dual Infection/Competition Assay Shows a Correlation between Ex Vivo Human Immunodeficiency Virus Type 1 Fitness and Disease Progression’, Journal of Virology, 74: 9222–33. 10982369 10.1128/jvi.74.19.9222-9233.2000 PMC102121

[vez029-B57] Ross T. M., Oran A. E., Cullen B. R. (1999) ‘Inhibition of HIV-1 Progeny Virion Release by Cell-Surface CD4 Is Relieved by Expression of the Viral Nef Protein’, Current Biology, 9: 613–21. 10375525 10.1016/s0960-9822(99)80283-8

[vez029-B58] Schaefer M. R. et al (2008) ‘HIV-1 Nef Targets MHC-I and CD4 for Degradation via a Final Common β-COP–Dependent Pathway in T Cells’, PLoS Pathogens, 4: e1000131. 18725938 10.1371/journal.ppat.1000131 PMC2515349

[vez029-B59] Sella G., Hirsh A. E. (2005) ‘The Application of Statistical Physics to Evolutionary Biology’, Proceedings of the National Academy of Sciences of the United States of America, 102: 9541–6. 15980155 10.1073/pnas.0501865102 PMC1172247

[vez029-B60] Shahid A. et al (2015) ‘Consequences of HLA-B*13-Associated Escape Mutations on HIV-1 Replication and Nef Function’, Journal of Virology, 89: 11557–71. 26355081 10.1128/JVI.01955-15 PMC4645636

[vez029-B61] Shekhar K. et al (2013) ‘Spin Models Inferred from Patient Data Faithfully Describe HIV Fitness Landscapes and Enable Rational Vaccine Design’, Physical Review E, 88: 062705. 10.1103/PhysRevE.88.062705 PMC5260469 24483484

[vez029-B62] Song H. et al (2012) ‘Impact of Immune Escape Mutations on HIV-1 Fitness in the Context of the Cognate Transmitted/Founder Genome’, Retrovirology, 9: 89. 23110705 10.1186/1742-4690-9-89 PMC3496648

[vez029-B63] Swigut T. et al (2004) ‘Impact of Nef-Mediated Downregulation of Major Histocompatibility Complex Class I on Immune Response to Simian Immunodeficiency Virus’, Journal of Virology, 78: 13335–44. 15542684 10.1128/JVI.78.23.13335-13344.2004 PMC525019

[vez029-B64] Toyoda M. et al (2015) ‘Differential Ability of Primary HIV-1 Nef Isolates to Downregulate HIV-1 Entry Receptors’, Journal of Virology, 89: 9639–52. 26178998 10.1128/JVI.01548-15 PMC4542390

[vez029-B65] Ueno T. et al (2007) ‘Altering Effects of Antigenic Variations in HIV-1 on Antiviral Effectiveness of HIV-Specific CTLs’, Journal of Immunology 178: 5513–23. 10.4049/jimmunol.178.9.5513 17442933

[vez029-B66] Ueno T. et al (2008) ‘CTL-Mediated Selective Pressure Influences Dynamic Evolution and Pathogenic Functions of HIV-1 Nef’, The Journal of Immunology, 180: 1107–16. 18178851 10.4049/jimmunol.180.2.1107

[vez029-B67] Watkins R. L. et al (2013) ‘In Vivo Analysis of Highly Conserved Nef Activities in HIV-1 Replication and Pathogenesis’, Retrovirology, 10: 125. 24172637 10.1186/1742-4690-10-125 PMC3907037

[vez029-B68] Wright J. K. et al (2012) ‘Impact of HLA-B*81-Associated Mutations in HIV-1 Gag on Viral Replication Capacity’, Journal of Virology, 86: 3193–9. 22238317 10.1128/JVI.06682-11 PMC3302318

