# Supplementary figures and images for: Modelling and in vitro testing of the HIV-1 Nef fitness landscape
Source: Virus Evol. 2019 Aug 5;5(2):vez029. doi: 10.1093/ve/vez029 (PMC6680064; doi:10.1093/ve/vez029)

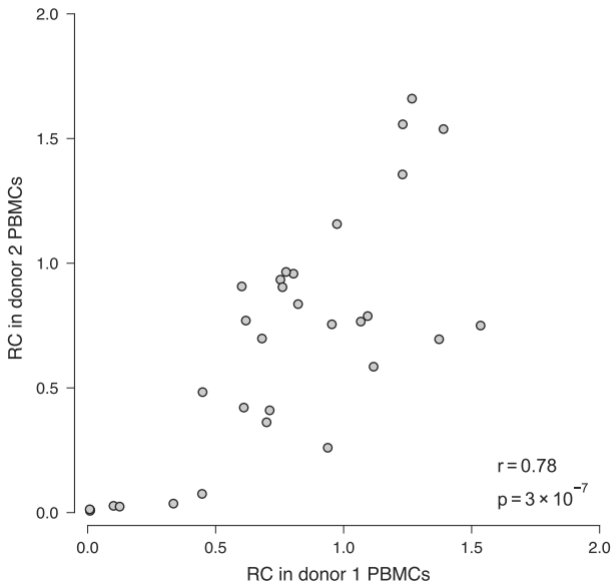

Supplement: vez029_Supplementary_Data [file vez029_supplementary_data.zip › figs2-rc-replicates.pdf]

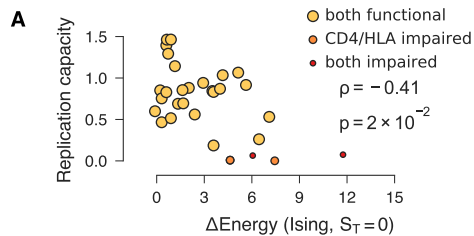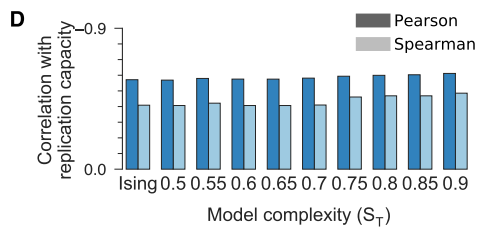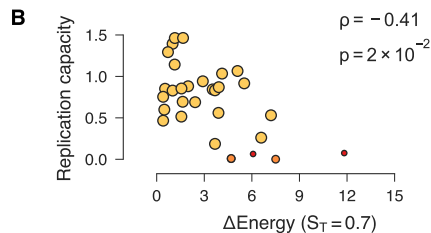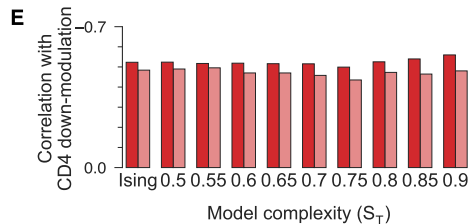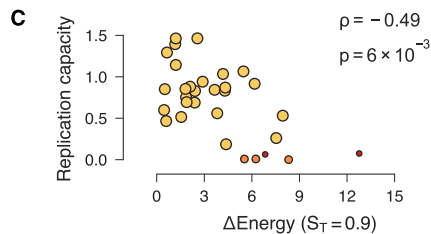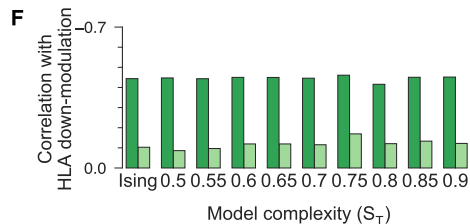

Supplement: vez029_Supplementary_Data [file vez029_supplementary_data.zip › figs3-correlation-all.pdf]

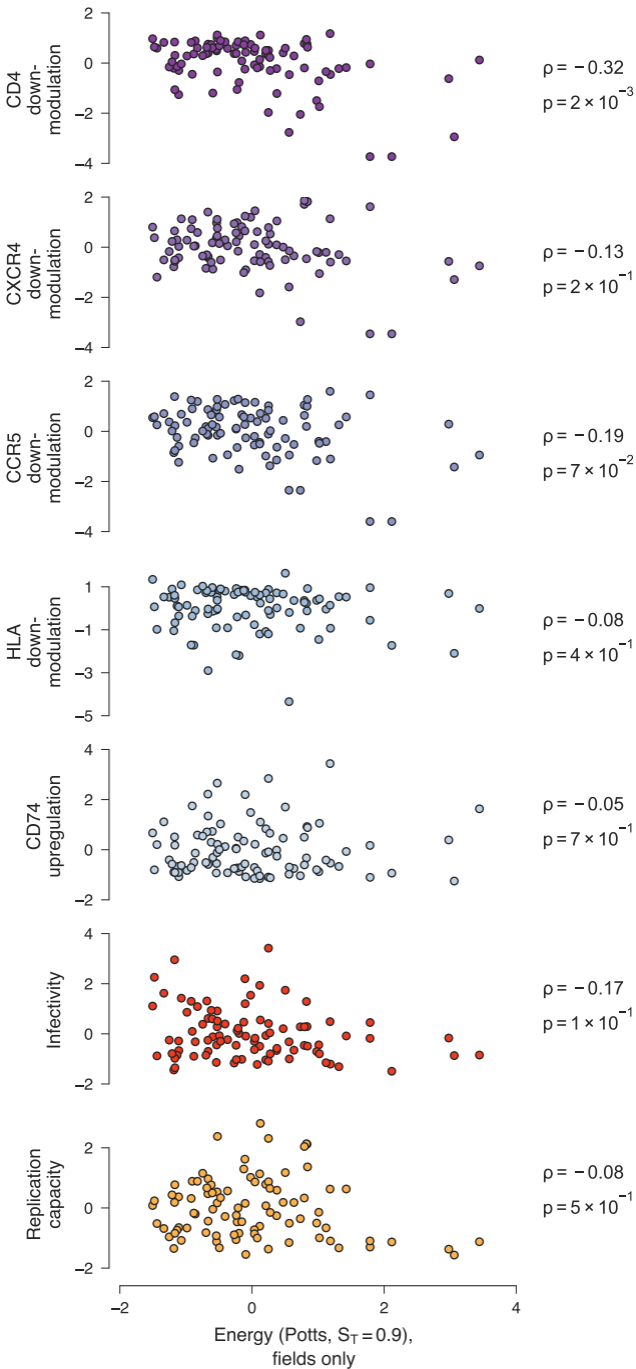

Supplement: vez029_Supplementary_Data [file vez029_supplementary_data.zip › figs4-function-vs-e-spearman-fields.pdf]

clade = C

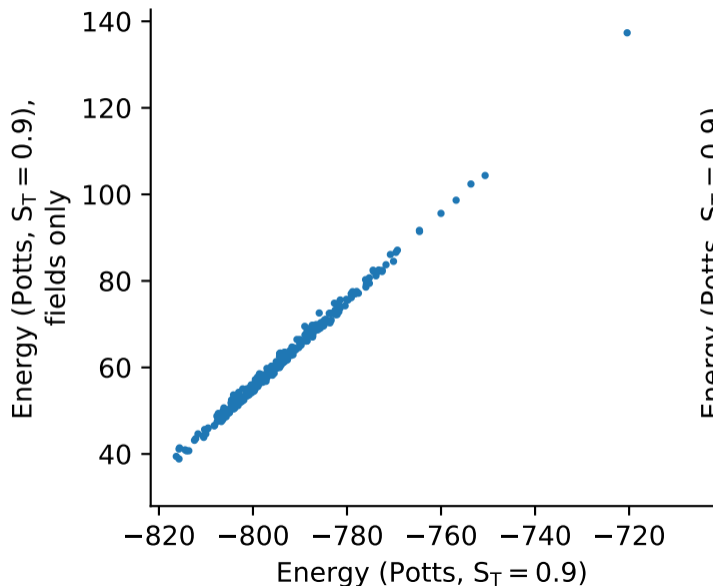

clade = B

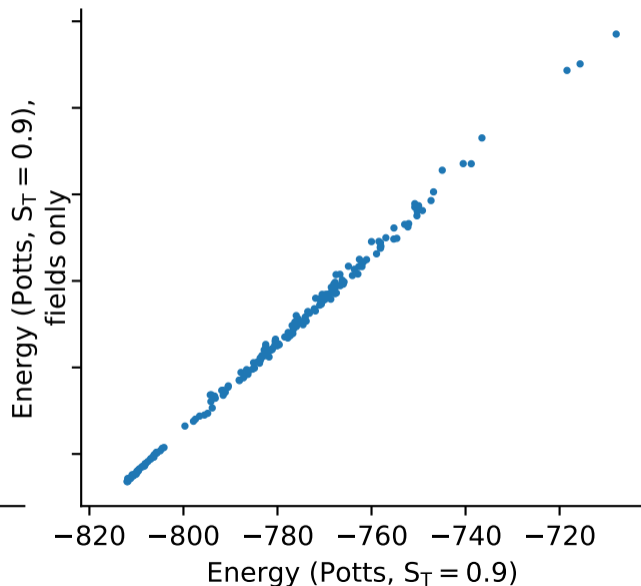

Supplement: vez029_Supplementary_Data [file vez029_supplementary_data.zip › figs5-E-vs-h.pdf]

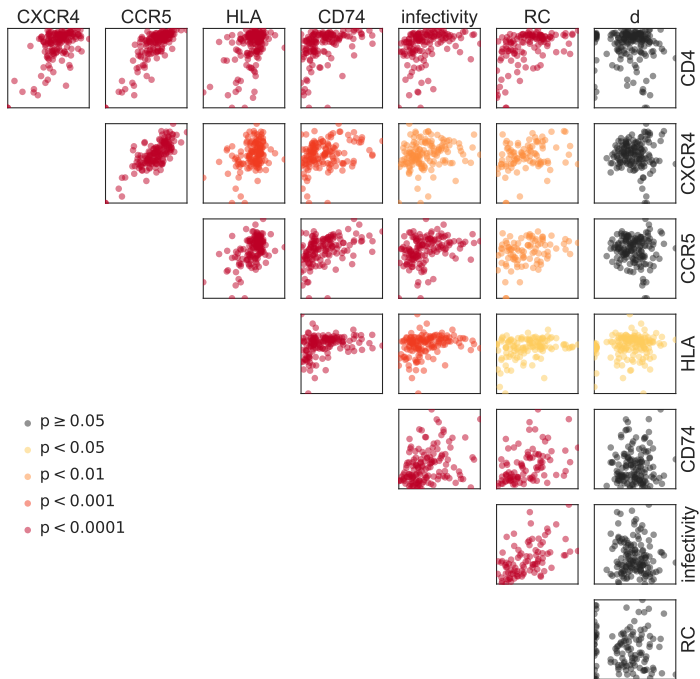

Supplement: vez029_Supplementary_Data [file vez029_supplementary_data.zip › figs6-functional-matrix-B (002).pdf]

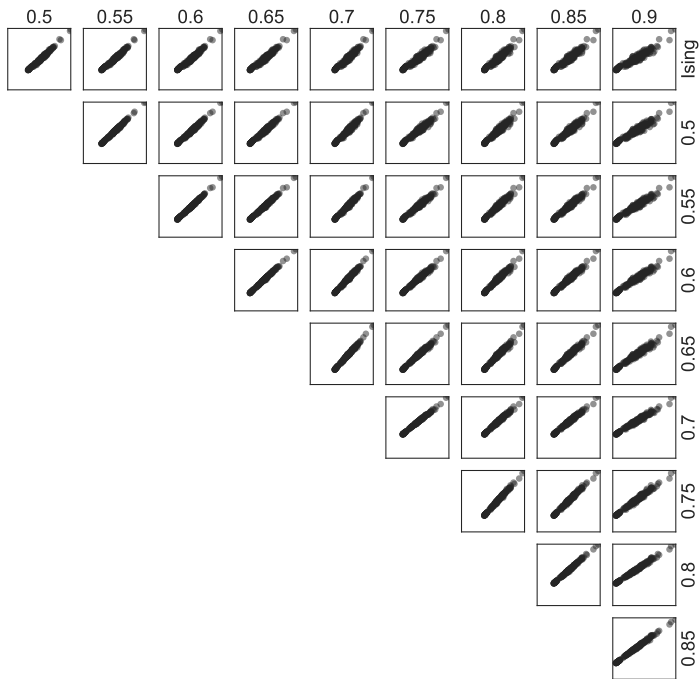

Supplement: vez029_Supplementary_Data [file vez029_supplementary_data.zip › figs1-e-matrix.pdf]
