# Supplementary material for: Modelling and in vitro testing of the HIV-1 Nef fitness landscape
Source: Virus Evol. 2019 Aug 5;5(2):vez029. doi: 10.1093/ve/vez029 (PMC6680064; doi:10.1093/ve/vez029)
Supplement: vez029_Supplementary_Data [file vez029_supplementary_data.zip › 2019-06-24-supplement.doc]

SUPPLEMENTAL MATERIAL FOR:

**Modeling and *in vitro* testing of the HIV-1 Nef fitness landscape**

**Authors:** John P. Barton^1,2*^, Erasha Rajkoomar^3*^, Jaclyn K. Mann^1^, Dariusz K. Murakowski^1^, Mako Toyoda^4^, Macdonald Mahiti^4^, Phillip Mwimanzi^4^, Takamasa Ueno^4,5^, Arup K. Chakraborty^1,2#^, Thumbi Ndung’u^2,3,6,7#^

**Affiliations: 1.** Department of Chemical Engineering, Massachusetts Institute of Technology, Cambridge, Massachusetts, United States of America. **2.** Ragon Institute of Massachusetts General Hospital, Massachusetts Institute of Technology and Harvard University, Boston, Massachusetts, United States of America. **3.** HIV Pathogenesis Programme, Doris Duke Medical Research Institute, Nelson R. Mandela School of Medicine, University of KwaZulu-Natal, Durban, South Africa. **4.** Center for AIDS research, Kumamoto University, Kumamoto, Japan. **5.** International Research Center for Medical Sciences (IRCMS), Kumamoto University, Kumamoto, Japan. **6**. Africa Health Research Institute, Durban, 4001, South Africa. **7**. Max Planck Institute for Infection Biology, Chariteplatz, D-10117 Berlin, Germany.

.

#Address correspondence to: Thumbi Ndung’u, [ndungu@ukzn.ac.za](mailto:ndungu@ukzn.ac.za)

Arup Chakraborty, arupc@mit.edu

***** These authors contributed equally to this work.

Supplementary Methods

Sequence data and processing. We downloaded a multiple sequence alignment (MSA) of HIV-1 clade B Nef protein sequences from the Los Alamos National Laboratory HIV sequence database (www.hiv.lanl.gov). The MSA were processed to remove insertions relative to the HXB2 reference sequence. We excluded sequences labeled as “problematic” in the database, and sequences with gaps or ambiguous amino acids present at >5% of residues were removed from the MSA. The remaining ambiguous amino acids were imputed with simple mean imputation. In total the MSA then contained 11354 sequences from 3300 unique individuals. Separately, we repeated this procedure for clade C protein sequences; the resulting MSA contained 6469 sequences from 1656 unique individuals.

We represent each sequence in the MSA can as a vector of variables ***z***={*z*_1_, z_2_, …, *z_N_*}, *z_i_*∈{A, R, …, V, -}, where *N*=206 is the length of the protein sequence. Each of the *z_i_* represents the amino acid (or gap) present at residue *i* in the sequence. The possible values of the *z_i_* are referred to as states. Our goal is to infer a model that accurately describes the distribution of HIV-1 Nef sequences circulating in the population, of which the sequences in the MSA are a sample. To describe this distribution we focus on the lowest moments: the frequency of each state at each residue, and the frequency of each pair of states at each pair of residues. These are

$$p_{i}^{*}(a)=\frac{1}{W}\sum_{k=1}^{B} w_{k}\delta(z_{i}^{(k)},a), p_{ij}^{*}(a,b)=\frac{1}{W}\sum_{k=1}^{B} w_{k}\delta(z_{i}^{(k)},a)\delta(z_{j}^{(k)},b) .$$

Here *k* is an index running from 1 to *B* used to label each sequence in the MSA, and *B* is the total number of sequences in the MSA. The function *δ* is the Kronecker *δ* function,

$$\delta(a,b)=\left\{\begin{matrix} 0 \text{if} a\neq b \\ 1 \text{if} a=b \end{matrix} .\right.$$

To prevent multiple sequences obtained from the same individual from biasing the sequence distribution, we weight the contribution of each sequence labeled *k* in the MSA by a factor *w_k_*, which is set equal to one divided by the total number of sequences in the MSA obtained from the same individual from whom the sequence labeled *k* was extracted. This ensures that the total weight of the sequences from each individual is equal. The normalizing factor *W* in equation is then the number of unique individuals from whom the sequences in the MSA were obtained, *W*=∑*_k_w_k_*. Following standard notation in statistical physics, we refer to the *p_i_**(*a*) and *p_ij_**(*a*,*b*) given in equation as correlations.

**Maximum entropy inference.** The “least biased” model capable of reproducing the observed correlations, interpreted as the model that maximizes the entropy of the sequence distribution, is a standard model in statistical physics referred to as the Potts model. In this model the probability of observing a sequence **z** is

$$P(\mathbf{z})=\frac{\exp\left(-E(\mathbf{z})\right)}{Q}, E(\mathbf{z})=-\sum_{i=1}^{N} h_{i}(z_{i})-\sum_{i=1}^{N-1} \sum_{j=i+1}^{N} J_{ij}(z_{i},z_{j}) .$$

*E*(**z**) is referred to as the energy of the sequence **z**, and

$$Q=\sum_{\mathbf{z}} \exp\left(-E(\mathbf{z})\right)$$

is a normalizing factor that guarantees that the probabilities of all sequences sum to one. The sum in equation is over all sequences of length *N*.

The parameters *h_i_*(*a*)*, J_ij_*(*a*,*b*) in equation must be chosen so that the Potts model correlations

$$p_{i}(a)=\sum_{\mathbf{z}} \delta(z_{i},a)\frac{\exp\left(-E(\mathbf{z})\right)}{Q}, p_{ij}(a,b)=\sum_{\mathbf{z}} \delta(z_{i},a)\delta(z_{j},b)\frac{\exp\left(-E(\mathbf{z})\right)}{Q} ,$$

are equal to their counterparts estimated from the MSA, given in equation . The problem of determining the *h_i_*(*a*)*, J_ij_*(*a*,*b*) parameters from the measured correlations is referred to as the inverse Potts problem. The solution is given by the set of parameters that maximize the log-likelihood function

However, this function cannot be directly maximized numerically because this requires evaluating *Q*, which involves a sum over a number of terms that is exponential in *N* (see equation ).

To solve this problem, we applied an extension of the adaptive cluster expansion method, described in ref. [1], with computational details in refs. [2,3]. For further details on implementation and code, see http://github.com/johnbarton/ACE and references therein.

**Sequence compression.** Even with the use of sophisticated algorithms, solving the inverse Potts problem remains a challenging computational task, due in part to the large number of parameters in the model. Overall, the model contains *N*(*q*-*1*) (*N*(*q*-1)+1) / 2 parameters, where *q* is the number of states (assuming here that this number is the same for each residue). Choosing *q*=21 for the 20 possible amino acids plus one gap state, we would require nearly 8.5 million variables to parameterize the Potts model for Nef.

Fortunately, it is not necessary to explicitly model all possible amino acids at each residue in order to characterize the sequence distribution. Here we adaptively adjust the number of states modeled at each residue based on the frequencies with which different amino acids are observed there in the MSA. Our procedure for choosing the number of states *q_i_* at each residue *i* is as follows. First, we order the amino acids at residue *i* according to how frequently they are observed in the MSA, with

$$p_{i}^{*}(a_{1})\geq p_{i}^{*}(a_{2})\geq\ldots\geq p_{i}^{*}(a_{21})\geq0 .$$

The Shannon entropy of the distribution of amino acids at this residue is

$$S=-\sum_{j=1}^{21-1} p_{i}^{*}(a_{j})\log p_{i}^{*}(a_{j})-(1-\sum_{j=1}^{21-1} p_{i}^{*}(a_{j}))\log(1-\sum_{j=1}^{21-1} p_{i}^{*}(a_{j})) ,$$

since the sum of the *p_i_**(*a*) over all states *a* must be equal to one. We then set *q_i_* equal to the smallest integer *q* such that

$$S_{q}=-\sum_{j=1}^{q-1} p_{i}^{*}(a_{j})\log p_{i}^{*}(a_{j})-(1-\sum_{j=1}^{q-1} p_{i}^{*}(a_{j}))\log(1-\sum_{j=1}^{q-1} p_{i}^{*}(a_{j}))\geq S_{T}\times S .$$

That is, we choose a number of states *q_i_* such that the reduced representation captures at least a fraction *S_T_* of the full entropy of the distribution of amino acids at that residue. The *q_i_*−1 most frequently observed amino acids at that residue each map to particular Potts states. All the remaining, less frequently observed amino acids map to a single aggregate state. Here we considered a range of possible values for *S_T_*, from 0 (i.e. a two-state or Ising model) to 0.9.

**Subgroup analysis of correlations between E values and Nef function.** In the main text, we examined the association between E values and Nef function for patient-derived clade B Nef sequences. We also performed the same tests on subsets of the data drawn from acute (n=49), chronic (n=46), and controller (n=45) patients. The overall pattern of correlations was largely consistent across these cohorts, though replication capacity measurements were not available for acute stage patients so these values could not be compared. Generally, correlations between E values and functional measurements were stronger for sequences from chronic patients than for acute. In particular, correlations between E and infectivity were substantially stronger for chronic (r=−0.47, p=1×10^−3^) patients than for acute (r=−0.15, p=3×10^−1^). This is notable because the sequences that we use to construct our landscape come overwhelmingly from chronic stage patients. Relationships between E values and function for sequences from controllers were similar to those from chronic stage patients, but with lower correlations with infectivity and higher correlations with replication capacity.

The same overall pattern is observed if these tests are performed for all subtype B sequences with measured CD4 and HLA-I down-modulation capabilities and replication capacities (r=0.49, p=2×10^-8^ for CD4 down-modulation and replication capacity; r=0.27, p=2×10^-3^ for HLA-I down-modulation and replication capacity; see Fig. S6).

Supplementary References

1.	Cocco S, Monasson R. Adaptive Cluster Expansion for Inferring Boltzmann Machines with Noisy Data. Phys Rev Lett. 2011;106: 090601. doi:10.1103/PhysRevLett.106.090601

2.	Barton JP, Cocco S. Ising models for neural activity inferred via selective cluster expansion: structural and coding properties. J Stat Mech. 2013;2013: P03002. doi:10.1088/1742-5468/2013/03/P03002

3.	Barton JP, De Leonardis E, Coucke A, Cocco S. ACE: adaptive cluster expansion for maximum entropy graphical model inference. Bioinformatics. 2016;32: 3089–3097.

4.	Steiger JH. Tests for comparing elements of a correlation matrix. Psychological bulletin. American Psychological Association; 1980;87: 245.

**Supplementary tables**

***_Table S1 | Pairwise comparison of statistical significance between replicative capacity – energy correlations using energy values from models with different values of ST_***

| *S_T_*=0 | *S_T_*=0.50 | *S_T_*=0.55 | *S_T_*=0.60 | *S_T_*=0.65 | *S_T_*=0.70 | *S_T_*=0.75 | *S_T_*=0.80 | *S_T_*=0.85 | *S_T_*=0.90 |  |
| --- | --- | --- | --- | --- | --- | --- | --- | --- | --- | --- |
| 1.000 | 0.715 | 0.630 | 0.826 | 0.836 | 0.619 | 0.452 | 0.353 | 0.267 | 0.155 | *S_T_*=0 |
|  | 1.000 | 0.497 | 0.718 | 0.729 | 0.540 | 0.372 | 0.299 | 0.217 | 0.117 | *S_T_*=0.50 |
|  |  | 1.000 | 0.733 | 0.714 | 0.879 | 0.555 | 0.476 | 0.376 | 0.227 | *S_T_*=0.55 |
|  |  |  | 1.000 | 0.826 | 0.201 | 0.427 | 0.339 | 0.247 | 0.126 | *S_T_*=0.60 |
|  |  |  |  | 1.000 | 0.134 | 0.414 | 0.331 | 0.240 | 0.122 | *S_T_*=0.65 |
|  |  |  |  |  | 1.000 | 0.585 | 0.465 | 0.361 | 0.200 | *S_T_*=0.70 |
|  |  |  |  |  |  | 1.000 | 0.740 | 0.579 | 0.309 | *S_T_*=0.75 |
|  |  |  |  |  |  |  | 1.000 | 0.656 | 0.302 | *S_T_*=0.80 |
|  |  |  |  |  |  |  |  | 1.000 | 0.409 | *S_T_*=0.85 |
|  |  |  |  |  |  |  |  |  | 1.000 | *S_T_*=0.90 |

Correlations between energy and replicative capacity were compared for synthetic subtype B mutants to look for significant differences with different entropy threshold values *S_T_* using Steiger’s Z test [4]. None of the pairwise comparisons are significant at *p*<0.05.

**Table S2 | Quantile regression of normalized functional measurements against Ising and Potts model E for natural subtype** B sequences

| **Predictor** | **Coefficient (Ising)** | **p value (Ising)** | **Coefficient (Potts, S_T_=0.9)** | **p value (Potts, S_T_=0.9)** |
| --- | --- | --- | --- | --- |
| **CD4 down-modulation** | -0.380 | 0.020 | -0.532 | 0.0004 |
| **HLA down-modulation** | 0.041 | 0.728 | 0.082 | 0.438 |
| **CXCR4 down-modulation** | -0.143 | 0.348 | -0.005 | 0.971 |
| **CCR5 down-modulation** | 0.029 | 0.889 | -0.008 | 0.965 |
| **CD74 upregulation** | 0.146 | 0.217 | 0.189 | 0.079 |
| **Infectivity** | -0.185 | 0.137 | -0.264 | 0.020 |
| **Replication capacity** | 0.158 | 0.203 | 0.230 | 0.041 |

Ising: pseudo R^2^ = 0.14; Potts: pseudo R^2^ = 0.19.

**Supporting Information Figures**

**Fig. S1. Nef mutant energies computed from models with different values of *S_T_* show excellent overall agreement.** Here we show a pairwise comparison between the energies of Nef sequences considered here for all values of *S_T_*. The energies clearly exhibit strong agreement, even between the models with the most extreme differences in complexity (*S_T_*=0 (Ising) versus *S_T_*=0.9).

**Fig. S2. Replication capacities (RC) for Nef mutants are broadly consistent when measured using PBMCs from two different donors.** Replication capacities measured in different PBMCs exhibit high correlation. Notably, however, variation in replication capacities can be large, in particular for viruses that replicate robustly. Points represent the mean of quadruplicate measurements (displaying an average standard deviation of 24.6%).

**Fig. S3. E values from models of all complexities are negatively correlated with *in vitro* functional capacity of mutant viruses.** (A-C) Correlation between replication capacities (RC) of mutant viruses and E values computed from models of different complexities (A, *S_T_*=0; B, *S_T_*=0.7; C, *S_T_*=0.9). Mutant Nef sequences with substantially impaired capacity to down-modulate CD4 or HLA (<80% of reference) are indicated by smaller points. These impaired Nef sequences also have very low replication capacities. RC of the mutant viruses is normalised relative to the wild-type virus. (D) Pearson and Spearman correlations between RC and E values as a function of model complexity. (E) Correlations between CD4 down-modulation capability and E values display a similar pattern of correlation as for RC. (F) Correlations between HLA down-modulation capability and E values are typically weak, but few mutant Nef sequences display significant impairment of HLA down-modulation.

**Fig. S4. Relationships between *in vitro* Nef functional measurements and E values from models with fields only.** Potts model energies are independently associated with a number of Nef functional measurements for natural subtype B sequences. This figure is analogous to Figure 5 in the main text, but here the Potts model does not include couplings *J_ij_*(*z_i_*, *z_j_*). Thus, in these models all sites are independent. For this data, we observe little difference between the independent site models and full models including all couplings.

**Fig. S5. The variation in E values for Nef sequences is dominated by contributions from the fields.** Plot of E values computed with and without couplings for all clade C and clade B Nef sequences considered in this study. Note that only *differences* in E values are physical, so the shift between E values with and without fields has no effect on predictions. E values cover a considerable range, the great majority of which can be attributed to the effects of fields alone.

**Fig. S6. Comparison of Nef functional measurements for natural and synthetic subtype B sequences.** Here we show a pairwise comparison between the Nef CD4 down-modulation, CXCR4 down-modulation, CCR5 down-modulation, HLA down-modulation, CD74 upregulation, infectivity, replication capacity (RC), and phylogenetic distance from the subtype B consensus sequence measurements for subtype B sequences (d). Scatter points in comparative subplots are colored according to the p value of their Spearman correlations.
